# Spatiotemporally restricted arenavirus replication induces immune surveillance and type I interferon-dependent tumour regression

**DOI:** 10.1038/ncomms14447

**Published:** 2017-03-01

**Authors:** Halime Kalkavan, Piyush Sharma, Stefan Kasper, Iris Helfrich, Aleksandra A. Pandyra, Asmae Gassa, Isabel Virchow, Lukas Flatz, Tim Brandenburg, Sukumar Namineni, Mathias Heikenwalder, Bastian Höchst, Percy A. Knolle, Guido Wollmann, Dorothee von Laer, Ingo Drexler, Jessica Rathbun, Paula M. Cannon, Stefanie Scheu, Jens Bauer, Jagat Chauhan, Dieter Häussinger, Gerald Willimsky, Max Löhning, Dirk Schadendorf, Sven Brandau, Martin Schuler, Philipp A. Lang, Karl S. Lang

**Affiliations:** 1 Institute of Immunology, Medical Faculty, University Duisburg-Essen, 45122 Essen, Germany; 2 Department of Medical Oncology, West German Cancer Center, University Hospital Essen, University Duisburg-Essen, 45122 Essen, Germany; 3 Department of Dermatology, West German Cancer Center, University Hospital Essen, University Duisburg-Essen, 45122 Essen, Germany; 4 Department of Cardiothoracic Surgery, Cologne University Heart Center, Kerpener Strasse 62, 50937 Cologne, Germany; 5 Department of Dermatology/Allergology, Cantonal Hospital, Rorschacher Strasse 95, St. Gallen 9007, Switzerland; 6 Department of Virology, Technical University of Munich, Schneckenburgstrasse 8, 81675 Munich, Germany; 7 Institute of Molecular Immunology/Experimental Oncology, München Klinikum rechts der Isar, Technical University Munich, 81675 Munich, Germany; 8 Division for Virology, Medical University Innsbruck, Peter-Mayr-Strasse 4b, 6020 Innsbruck, Austria; 9 Institute of Virology, Düsseldorf University Hospital, Heinrich Heine University, Universitätsstrasse 1, 40225 Düsseldorf, Germany; 10 Department of Molecular Microbiology and Immunology, University of Southern California, Los Angeles, 90033 California, USA; 11 Institute of Medical Microbiology and Hospital Hygiene, University of Düsseldorf, Universitätsstrasse 1, 40225 Düsseldorf, Germany; 12 Ludwig Institute for Cancer Research, University of Oxford, Old Road Campus, Research Building, Old Road Campus, Headington, Oxford OX3 7DQ, UK; 13 Department of Gastroenterology, Hepatology and Infectious Diseases, University of Düsseldorf, Universitätsstrasse 1, 40225 Düsseldorf, Germany; 14 Institute of Immunology (Charité - University Medicine Berlin), 3125 Berlin, Germany; 15 German Cancer Research Center (DKFZ), 13125 Heidelberg, Germany; 16 Department of Rheumatology and Clinical Immunology, Charité—University Medicine Berlin and German Rheumatism Research Center (DRFZ), Charitéplatz 1, D-10117 Berlin, Germany; 17 German Cancer Consortium (DKTK), Partner Site University Hospital Essen, 45122 Essen, Germany; 18 Department of Otorhinolaryngology, West German Cancer Center, University Hospital Essen, University Duisburg-Essen, 45122 Essen, Germany; 19 Department of Molecular Medicine II, Medical Faculty, Heinrich Heine University, Universitätsstrasse 1, D-40225 Düsseldorf, Germany

## Abstract

Immune-mediated effector molecules can limit cancer growth, but lack of sustained immune activation in the tumour microenvironment restricts antitumour immunity. New therapeutic approaches that induce a strong and prolonged immune activation would represent a major immunotherapeutic advance. Here we show that the arenaviruses lymphocytic choriomeningitis virus (LCMV) and the clinically used Junin virus vaccine (Candid#1) preferentially replicate in tumour cells in a variety of murine and human cancer models. Viral replication leads to prolonged local immune activation, rapid regression of localized and metastatic cancers, and long-term disease control. Mechanistically, LCMV induces antitumour immunity, which depends on the recruitment of interferon-producing Ly6C^+^ monocytes and additionally enhances tumour-specific CD8^+^ T cells. In comparison with other clinically evaluated oncolytic viruses and to PD-1 blockade, LCMV treatment shows promising antitumoural benefits. In conclusion, therapeutically administered arenavirus replicates in cancer cells and induces tumour regression by enhancing local immune responses.

## Abstract (web summary)

Viruses are promising anti-tumour therapeutics due to induction of an immune response and/or oncolytic activity. Here, Kalkavan *et al*. show that LCMV replicates in tumour cells, without inducing cell lysis, and that its anti-tumour activity is largely mediated by recruitment of interferon-producing monocytes.

Effective treatment of advanced tumours remains a major challenge because of limited availability of tumour-specific agents and development of drug resistance. Next to chemotherapy and targeted therapies, immunotherapy is one promising approach to treat cancer^1^.

The immune system can directly attack tumour cells via antigen-specific cytotoxic CD8^+^ T cells, activated natural killer (NK) cells or antibody-mediated cytotoxicity^2^,^3^. In addition, cytokines such as tumour necrosis factor-α, interferon (IFN)-γ or type I IFN (IFN-I) can directly exert antiproliferative and pro-apoptotic effects on tumour cells, or indirectly, through modulation of the tumour microenvironment^1^,^4^. Despite the physiological existence of these potent anticancer effector molecules, neoplastic cells can survive and expand in immune-competent individuals. Escape from immune surveillance is mainly explained by limited immune activation or tumour-induced immunosuppression within the microenvironment^2^. Effective induction of strong and sustained immune activation at the tumour sites would therefore be a promising therapeutic approach against cancer.

Viruses have a very high capacity to activate the innate and adaptive immune system. This is mainly explained by three mechanisms. First, viruses introduce new antigens to the immune system, which are recognized by the host as foreign^5^. Second, viruses carry ligands for pattern recognition receptors, which trigger the innate immune system^5^. Third, viruses are usually drained from peripheral sites to the lymphatic system where they undergo spatiotemporally restricted replication in antigen-presenting cells and thereby specifically activate innate and adaptive immune cells^6^,^7^.

Arenaviruses are enveloped and pleiomorphic, with a diameter of 60–300 nm and two single-stranded RNA genome segments. The non-cytopathic arenaviruses propagate rapidly without directly harming susceptible tissues. Rather, it is the immune response against infected cells that may cause severe tissue damage and disease symptoms^8^,^9^. Arenaviruses can infect humans and disease outcome depends on the specific strain. Lassa virus and Junin virus are responsible for the Lassa and Argentine haemorrhagic fever, respectively^10^. In contrast, human infection with the arenavirus strains lymphocytic choriomeningitis virus (LCMV, strain WE) and Candid#1, which is a clinically applied vaccine virus to protect against Argentine haemorrhagic fever, is usually asymptomatic or causes nonspecific symptoms such as fever and malaise^11^,^12^. LCMV-WE induces a strong T-cell response, which can be antitumoural in cancer models^13^,^14^. Therefore, recombinant single-cycle LCMV is considered a vaccine virus with potential to immunize against tumour antigens^15^.

Here we investigate whether neoplastic cells can serve as natural reservoir for arenavirus replication and whether such replication can induce spatiotemporally restricted innate immunity, virus-specific and tumour-specific adaptive immune activation. We show that LCMV and Candid#1 preferentially replicate in cancer cells and induce immune surveillance resulting in IFN-I-dependent tumour regression.

## Results

### Arenavirus preferentially replicates in cancer cells

**Figure 1:**
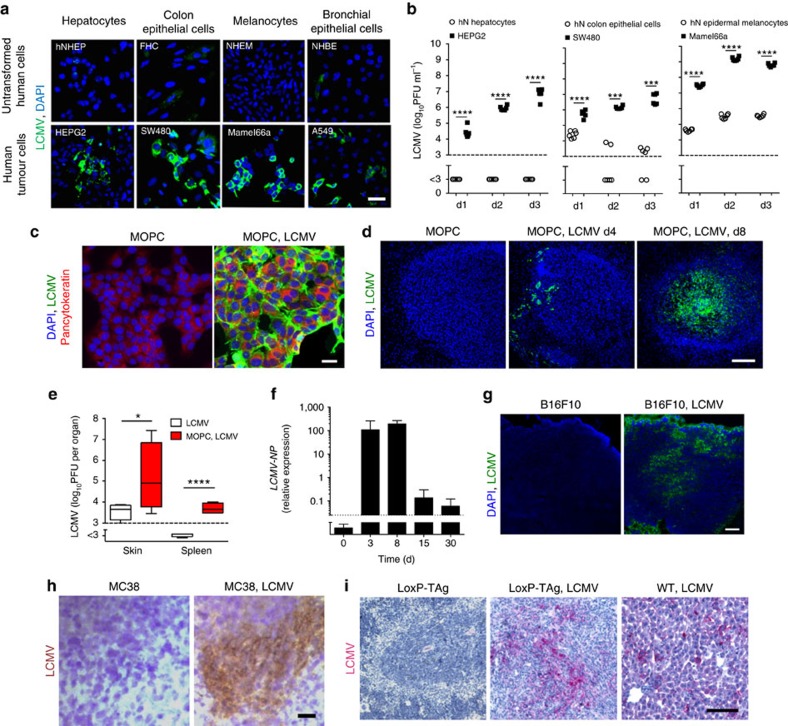
Arenavirus preferentially replicates and persists in cancer cells. (**a**) Immunofluorescence in tissue-matched human normal untransformed and tumour cells 2 days after LCMV infection (MOI 1) (*n*=3 per group). Scale bar, 20 μm. (**b**) Infectious virus particles in supernatants from human normal (hN) untransformed and tumour cells, which were infected with LCMV (MOI 1) and analysed on indicated days (*n*=6 per group). (**c**) Immunofluorescence of MOPC cells untreated or infected with LCMV (MOI 1) 72 h after infection (*n*=3/group). Scale bar, 20 μm. (**d**) Immunofluorescence of tumours in MOPC-tumour-bearing C57BL/6 mice (day −10) treated with or without 2 × 10^4^ PFU LCMV peritumourally (LCMV, green; 4,6-diamidino-2-phenylindole (DAPI), blue, *n*=3 per group). Scale bar, 200 μm. (**e**) Infectious virus particles in skin and spleen (day 8) of C57BL/6 mice or MOPC-tumour-bearing mice (day −3) treated with 2 × 10^4^ PFU LCMV subcutaneously (*n*=4 per group). (**f**) LCMV-NP RNA in tumours of MOPC-tumour-bearing C57BL/6 mice (day −10) treated with 2 × 10^4^ PFU LCMV intratumourally (*n*=8 per group). (**g**) Immunofluorescence of tumours (day 7, *n*=3) from B16F10-tumour-bearing C57BL/6 mice (day −3) treated with or without 2 × 10^4^ PFU LCMV peritumourally. Scale bar, 200 μm. (**h**) Immunohistochemistry of tumours (day 7, *n*=3) from MC38-bearing C57BL/6 mice (day −3) treated with (*n*=6) or without (*n*=7) 2 × 10^4^ PFU LCMV peritumourally. Scale bar, 200 μm. (**i**) Immunohistochemistry (day 6, *n*=3) of livers from LoxP-Tag-tumour-bearing or WT mice, which were treated with or without 2 × 10^6^ PFU LCMV systemically. Scale bar, 200 μm. Data are shown as mean±s.e.m. and analysed by unpaired Student's *t*-test. *P<0.05, ***P<0.001 and ****P<0.0001.

In recent times, we found that fast replication of arenavirus in dendritic cells led to massive activation of the innate and adaptive immune system^6^. As cancer cells are characterized by altered cell cycle, metabolism and translation^16^ relative to their normal counterparts, we wondered whether arenaviruses might preferentially replicate in tumour cells and whether this affects the antitumoural immune response. To explore the replication capacity of arenaviruses in tumour cells, we infected human cancer cells and the healthy parenchymal counterpart they originated from with the non-cytopathic LCMV. Primary hepatocytes, colon epithelial cells, melanocytes and bronchial epithelial cells showed limited LCMV replication as compared with malignant cells originating from these tissues (Fig. 1a,b). Having established that arenaviruses preferentially replicate in tumour cells, we next infected a tumorigenic cell line that is capable of forming tumours in a syngeneic setting using immune-competent mice. Specifically, we infected the murine squamous oropharynx carcinoma cell line (MOPC)^17^ with LCMV-WE *in vitro*. Within 72 h, all cancer cells revealed LCMV replication without affected cell survival (Fig. 1c and Supplementary Fig. 1). Next, we established palpable tumours in C57BL/6 mice by subcutaneous injection of MOPC cells and infected the mice with 2 × 10^4^ plaque-forming unit (PFU) LCMV peritumourally. Immunofluorescence revealed viral replication within these tumours, but not in other regions of the skin (Fig. 1d). The presence of subcutaneous MOPC tumours significantly increased local and splenic virus propagation in C57BL/6 mice (Fig. 1e) and virus persisted in tumours for more than 30 days (Fig. 1f). To generalize this phenomenon, we analysed the replication of LCMV in a broad panel of other murine cancer models. Peritumoural injection of LCMV in subcutaneously established B16F10 melanoma^18^ and MC38 colon cancer^19^, as well as intravenous administration of LCMV in endogenously hepatocellular carcinoma bearing LoxP-TAg^20^ resulted in intratumoural propagation of LCMV (Fig. 1g–i).

### B-Myb promotes arenavirus replication

Lack of IFN-I receptor and deregulated IFN signalling is proposed to be one mechanism accounting for enhanced viral replication and IFN-unresponsiveness in cancer cells^21^,^22^. However, analysis of the Cancer Genome Atlas (TCGA) database revealed high expression of *Ifnar1* and *Ifnar2* in a range of cancer entities (Supplementary Fig. 2a) and human cancer cell lines showed expression of *Ifnar1* and *Ifnar2* (Supplementary Fig. 2b). To test whether human cancer cell lines can respond to some of the many murine IFN-I in a xenograft system, we treated Sw480 cells and HeLa cells with different IFN-I subtypes *in vitro*. Sw480 and HeLa cells responded to murine IFN-α2 and human IFN-α4 but not to murine IFN-α4 (Supplementary Fig. 2c). Moreover, all the murine cancer cells tested in our *in vivo* studies expressed *Ifnar1* (Supplementary Fig. 2d). In comparison with MC38, B16F10, LoxP-TAg and MT/ret, MOPC cells showed highest expression levels (Supplementary Fig. 2d).

We hypothesized that the fast cell cycle and metabolism of cancer cells promote replication of arenavirus and thereby explains the fast propagation of LCMV in cancer cells. Recent reports show that the expression of the cell cycle kinase CDK2 is associated with increased HIV replication in myeloid and lymphoid cells^23^. CDK2 phosphorylates B-Myb, which enhances the transcription of a number of genes involved in cell proliferation and metabolism^24^,^25^. To explore whether B-Myb (*Mybl2*) is one factor in cancer cells contributing to enhanced arenavirus replication, we studied *Mybl2* expression and phosphorylation in cancer tissues and non-malignant controls. Indeed, tumours expressed higher *Mybl2* RNA levels and exhibited increased B-Myb phosphorylation (Supplementary Fig. 2e,f). The presence of B-Myb correlated with higher expression of known viral host factors in MOPC tumours than control tissue (Supplementary Fig. 2g). In line, knockdown of B-Myb limited LCMV replication (Supplementary Fig. 2h). Therefore, we concluded that B-myb overexpression is one viral susceptibility factor in cancer cells.

### Arenavirus replication leads to tumour regression

**Figure 2:**
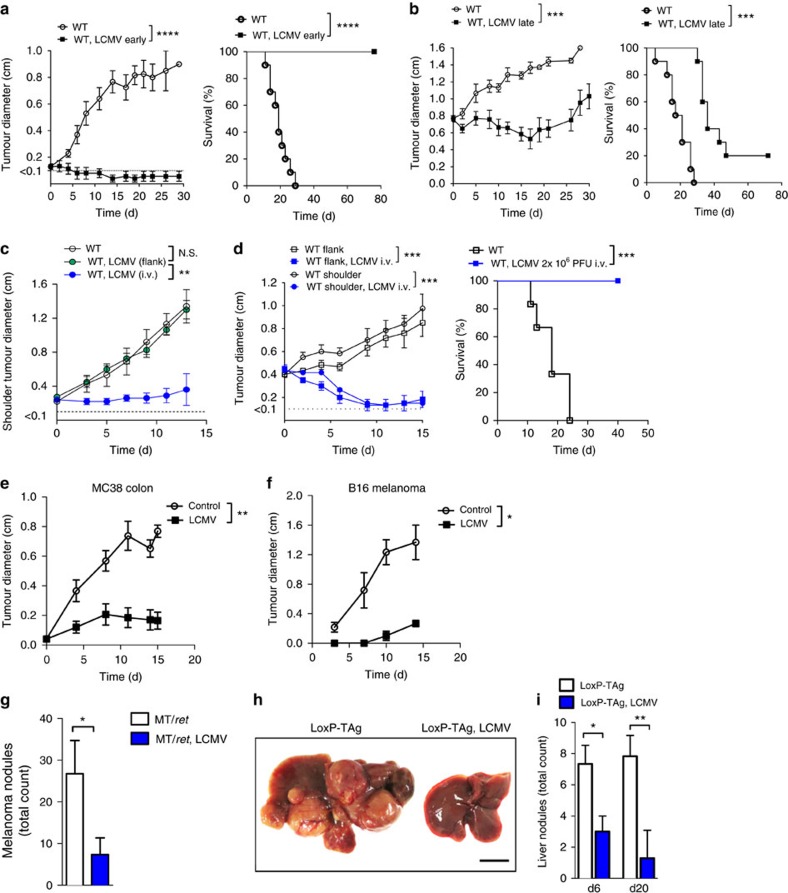
Persistence of arenavirus leads to tumour regression. (**a**) Tumour diameter and survival of MOPC-tumour-bearing mice (day −3) treated with or without 2 × 10^4^ PFU LCMV peritumourally (*n*=10 per group, three experiments pooled). (**b**) Tumour diameter and survival of MOPC-tumour-bearing mice (day −10) treated with or without 2 × 10^4^ PFU LCMV intratumourally (*n*=10 per group, three experiments pooled). (**c**) Tumour diameter of the shoulder tumour from C57BL/6 mice receiving simultaneously subcutaneously 5 × 10^5^ MOPC cells in the flank and shoulder (day −3), treated without (*n*=5) or with 2 × 10^4^ PFU LCMV given into the flank (*n*=4) or intravenously (*n*=5). (**d**) Tumour diameters (shoulder and flank) and survival of WT mice bearing MOPC tumours simultaneously in the shoulder and flank, treated with or without 2 × 10^6^ PFU LCMV intravenously on day 0 (*n*=6 per group). (**e**) Tumour diameter from MC38-bearing C57BL/6 mice (day −3) treated with (*n*=6) or without (*n*=7) 2 × 10^4^ PFU LCMV peritumourally. (**f**) Tumour diameters from B16F10-tumour-bearing C57BL/6 mice (day −3) treated with or without 2 × 10^4^ PFU LCMV peritumourally (*n*=6 per group). (**g**) Number of melanomas (day 15) in MT/*ret* mice treated with (*n*=3) or without (*n*=4) 2 × 10^4^ PFU LCMV systemically. (**h**,**i**) Representative picture (**h**, day 6, *n*=3) and quantification of tumour nodules (**i**, day 6 *n*=3, day 20 *n*=6) of/in the livers from LoxP-Tag-tumour-bearing or WT mice, which were treated with or without 2 × 10^6^ PFU LCMV systemically. Scale bar, 0.5 cm. Data are shown as mean±s.e.m. and analysed by unpaired Student's *t*-test. *P<0.05, **P<0.01, ***P<0.001 and ****P<0.0001.

Next we wondered whether LCMV replication in tumour cells influences tumour progression. We infected C57BL/6 mice bearing small tumours with LCMV peritumourally. Untreated control tumours grew robustly and mice succumbed to the tumour within 30 days (Fig. 2a). Peritumoural LCMV injection completely abolished tumour growth and mice survived relapse-free for an observational time of 80 days (Fig. 2a), without virus- or tumour-related symptoms. In mice bearing very advanced MOPC tumours, LCMV infection induced considerable tumour regression and significantly prolonged survival (Fig. 2b). To explore the activity of LCMV virotherapy in metastatic cancers, we compared local and systemic virus administration in mice bearing simultaneous MOPC tumours in the left shoulder and the right flank. Peritumoural injection of LCMV in the right flank resulted in virus replication only in the flank (Supplementary Fig. 3a). Systemically administered LCMV reached both tumour sites (Supplementary Fig. 3a). In agreement, intravenous LCMV application, but not local LCMV injection into the flank tumour, induced regression of distant shoulder tumours (Fig. 2c). Conclusively, control of multiple tumour sites and prolonged survival of mice with advanced stage metastatic tumours can be achieved by systemic LCMV therapy (Fig. 2d). Next, we analysed the antitumoural activity of LCMV against a broad panel of murine cancer models including MC38 colon cancer^19^, B16F10 melanoma^18^, the endogenous hepatocellular carcinoma model LoxP-TAg^20^ and spontaneous MT/*ret* melanoma model^26^. Peritumoural injection of LCMV limited the growth of subcutaneous MC38 (Fig. 2e) and B16F10 melanoma tumours (Fig. 2f). To test the activity of LCMV virotherapy on spontaneously developing malignant melanomas in the MT/*ret* mouse model^26^, LCMV was injected intravenously once cutaneous tumours became palpable. Strikingly, LCMV-infected MT/*ret* mice developed significantly less macroscopically visible tumour nodules than untreated mice (Fig. 2g). Next, we studied LCMV virotherapy in endogenously developing liver cancers. Nine-month-old LoxP-TAg mice harbouring palpable liver tumours either received an intravenous injection of LCMV (2 × 10^6^ PFU) or were left untreated. Intriguingly, 20 days after LCMV injection tumour nodules had largely disappeared (Fig. 2h,i). Consistent with these findings, serum liver enzyme levels, which reflect tumour burden in the liver, were significantly lower in LCMV-treated LoxP-TAg mice than in uninfected LoxP-TAg controls (Supplementary Fig. 3b).

### IFN-I was essential for tumour regression

**Figure 3:**
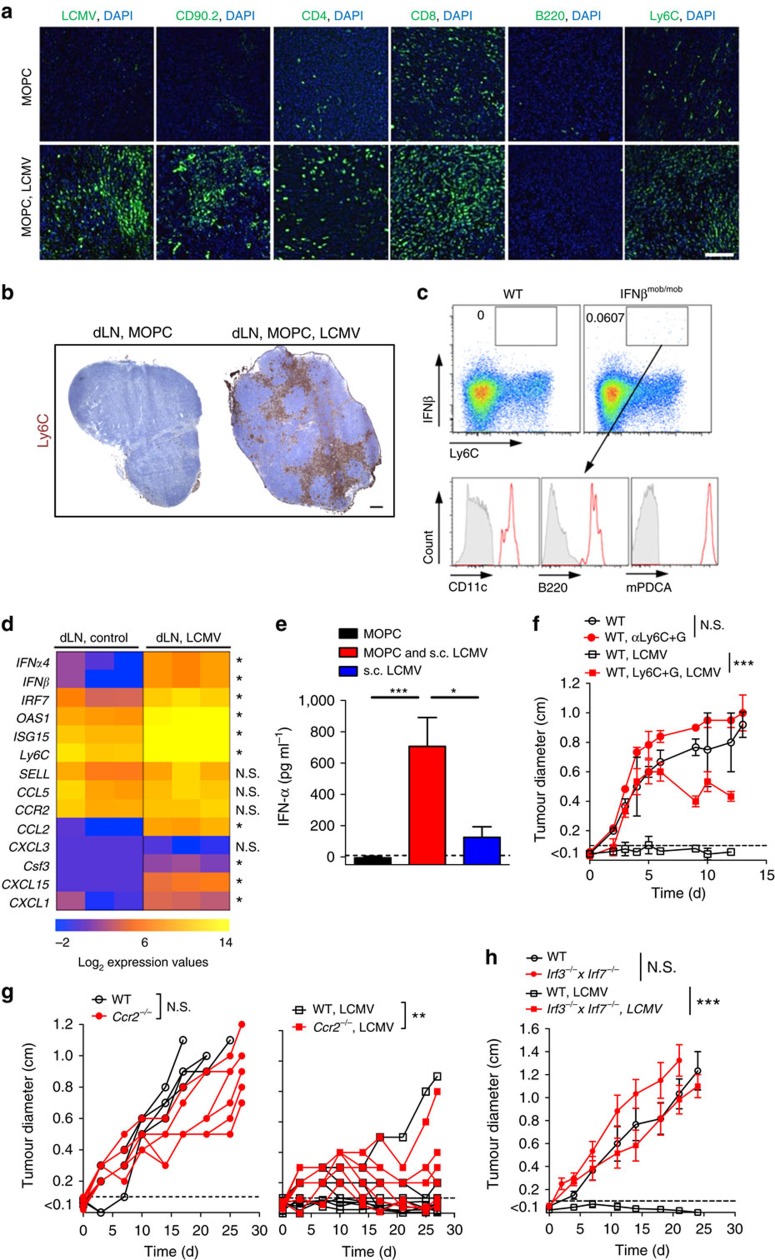
IFN-I was essential for arenavirus-induced tumour regression. (**a**) Immunofluorescence (day 10) of tumours from MOPC-tumour-bearing C57BL/6 mice (day −10) treated with or without 2 × 10^4^ PFU LCMV peritumourally (*n*=3/group). Scale bar, 200 μm. (**b**) Immunohistochemistry of dLNs from MOPC-tumour-bearing mice treated with 2 × 10^4^ PFU LCMV subcutaneously (*n*=3/group). Scale bar, 200 μm. (**c**) Representative FACS blots (day 2) from dLNs of MOPC-tumour-bearing C57BL/6 mice (day −3) and IFN-β-reporter mice (IFNβ^mob/mob^) treated with 2 × 10^4^ PFU LCMV peritumourally (day 0, *n*=4 per group). Grey area indicates isotype control. (**d**) qRT–PCR analysis of dLNs (day 3) from MOPC-tumour-bearing C57BL/6 mice (day −3) treated with or without 2 × 10^4^ PFU LCMV peritumourally (*n*=3 per group). (**e**) IFN-α serum ELISA (day 3) from MOPC-tumour-bearing C57BL/6 mice (day −3) treated with or without 2 × 10^4^ PFU LCMV (*n*=4 per group). (**f**) Tumour diameters of MOPC-tumour-bearing C57BL/6 mice (day −3) injected with or without anti-Ly6C+G antibody (200 μg, days −2, 2 and 7) and treated with (*n*=6 per group) or without (*n*=6 control; *n*=7 anti-Ly6C+G-dep.) 2 × 10^4^ PFU LCMV peritumourally (two experiments pooled). (**g**) Tumour diameters of MOPC-tumour-transplanted WT and *Ccr2*^*–/–*^ mice (day −3) treated with (*n*=9 per group) or without (*n*=6 per group) 2 × 10^4^ PFU LCMV peritumourally (two experiments pooled). (**h**) Tumour diameter from MOPC-tumour-bearing WT and *Irf3*^*–/–*^ *x Irf7*^*–/–*^ mice (day × 3) treated with or without 2 × 10^4^ PFU LCMV peritumourally (*n*=6 per group, two experiments pooled). Data are shown as mean±s.e.m. and analysed by unpaired Student's *t*-test. Survival is shown in Kaplan–Meier method and analysed by log-rank test. NS, nonsignificant; *P<0.05, **P<0.01 and ***P<0.001.

To dissect whether LCMV replication or LCMV-induced immune infiltration impacts tumour growth, we studied tumour growth in *Map3k14*^*aly/aly*^ mice, which have defective nuclear factor-κB signalling and therefore lack lymph nodes and immune functions^27^. LCMV-induced tumour regression was dependent on *Map3k14* (Supplementary Fig. 4), suggesting that immune infiltration is crucial. Indeed, LCMV replication induced a dense infiltrate composed of T cells (CD90.2, CD4 and CD8) and inflammatory Ly6C^+^ monocytes (Fig. 3a). Strong infiltrates of Ly6C^+^ monocytes were detected in draining lymph nodes (dLNs) of LCMV-treated subcutaneous MOPC tumours (Fig. 3b). Monocytes have the plasticity to execute diverse effector functions and are therefore equipped to inhibit tumour growth^28^. As inflammatory monocytes also produce IFN-I after innate sensing^29^, we postulated that LCMV-induced IFN-I secretion in Ly6C^+^ monocytes impacts tumour growth. To analyse IFN-I production in Ly6C^+^ monocytes, we established MOPC tumours in IFN-β reporter mice (IFNβ^mob/mob^) and infected them with LCMV^30^. IFN-β-producing Ly6C^+^ monocytes were detected in the dLN of tumour-bearing mice after infection with LCMV (Fig. 3c). Ly6C^+^ monocytes were further characterized as mPDCA-1^hi^, CD11c^med^ and B220^med^ (Fig. 3c), suggesting that they were differentiated into IFN-I-producing cells^31^. Consistently, quantitative real-time PCR (qRT–PCR) analysis revealed high expression of IFN-I-associated genes in dLN after LCMV infection, and IFN-α serum levels were enhanced in LCMV-infected tumour-bearing mice (Fig. 3d,e). To dissect the role of monocytes and IFN-I on tumour regression, we studied tumour growth in mice depleted of myeloid cells *in vivo* using different antibodies. First, we treated mice with an antibody against the myeloid differentiation antigen Gr-1 (clone RB6-8C5), which depletes Ly6G^+^ and Ly6C^+^ cells^32^,^33^,^34^ (Supplementary Fig.5a). Another experimental group was treated with an antiLy6G antibody (clone 1A8), which only binds to Ly6G^+^ cells (Supplementary Fig. 5b). Depletion of Ly6C^+^ and Ly6G^+^ cells (monocytes and granulocytes) with clone RB6-8C5 abrogated LCMV-mediated tumour suppression (Fig. 3f), whereas depletion of Ly6G^+^ cells (granulocytes) with clone 1A8 alone had no impact on the antitumoural effect of LCMV (Supplementary Fig. 5c). This suggests that monocytes are centrally involved in LCMV-induced tumour regression. The role of monocytes was confirmed using *Ccr2*^*−/−*^ mice, which have reduced monocyte numbers^28^ and in which LCMV-mediated tumour suppression was significantly blunted (Fig. 3g). To corroborate the involvement of IFN-I in LCMV-mediated tumour regression, we used *Irf3*^*−/−*^*xIrf7*^*−/−*^ mice, which lack IFN-I induction after LCMV infection^35^. LCMV infection failed to suppress tumour growth in *Irf3*^*−/−*^*xIrf7*^*−/−*^ mice (Fig. 3h), supporting the functional importance of IFN-I for the antitumour effect of arenavirus infection. Analysis of T cells (Supplementary Fig. 6a), B cells (Supplementary Fig. 6b) and NK cells (Supplementary Fig. 6c,d) failed to reveal an impact of these immune cell subsets on LCMV-induced control of early stage tumours, suggesting that IFN-I acts independent of these cell subsets on tumour regression.

### Monocyte recruitment and IFN-I induction in human cancer

To gain further insights into the possible translation of arenaviruses to human cancer therapy we studied primary tumour biopsies from 34 patients with oropharyngeal cancers. Multigene expression analysis by qRT–PCR revealed a strong correlation between the expression of human monocytic markers *CD14* or *CD16* and diverse IFN-I-related genes such as *IFNB1*, *USP18* and *IRF7* (Supplementary Fig. 7a,b), indicating that human monocytic infiltrates can produce IFN-I within human tumour tissues. To investigate whether our observation is expandable to different human cancer types, we accomplished a gene-set enrichment analysis (GSEA) by using the human cancer TCGA database. Indeed, *CD14*^*+*^ cell populations were enriched for IFN-I-related genes (Supplementary Fig. 7c)^36^.

### Ambiguous role of CD8^+^ T cells in cancer virotherapy

**Figure 4:**
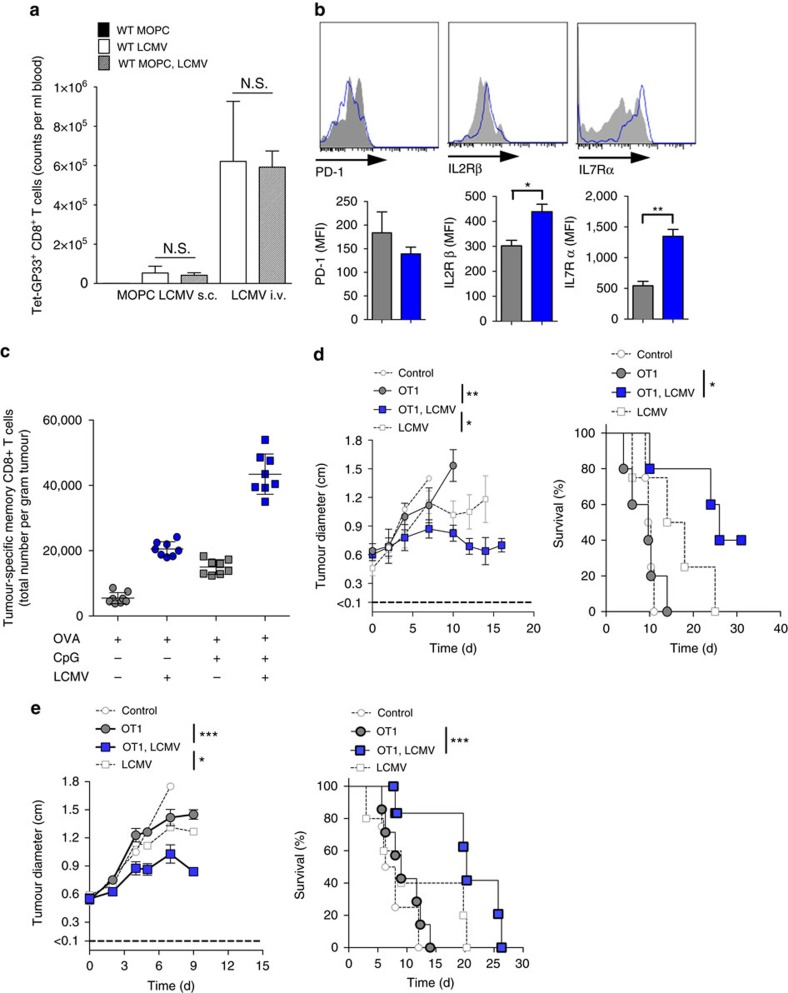
CD8^+^ T-cell activation contributes to arenavirus-mediated tumour regression. (**a**) LCMV-specific CD8^+^ T cells in the blood (day 8) of MOPC-tumour-bearing or control mice, which where infected with LCMV (*n*=3). (**b**) Expression of PD-1, IL-2Rβ and IL-7Rα on tumour-specific CD8^+^ T cells (OT1, day 8) of B16-OVA-tumour-bearing C57BL/6 mice (day −10) transferred with 5 × 10^6^ OT1 splenocytes (day −1) and additionally treated with (*n*=5) or without (*n*=3) LCMV (i.t. 2 × 10^4^ PFU). (**c**) Total numbers of tumour-specific (OVA), antigen-exposed (CD44^+^) CD8^+^ T cells (day 2) in tumours of B16-OVA-tumour-bearing C57BL/6 mice (day −13), which were vaccinated with ovalbumin (200 μg, day −3, i.v.) with or without CpG (20 μg, day −3, i.v.) and additionally treated with or without LCMV (5 × 10^5^ PFU, i.t., *n*=8). (**d**) Tumour diameter and survival of B16-OVA-tumour-bearing C57BL/6 mice (day −10) treated with (*n*=5 per group) or without (*n*=4 per group) 5 × 10^6^ OT1 splenocytes (day −1) and additionally treated with or without LCMV (i.t. 2 × 10^4^ PFU). (**e**) Tumour diameter and survival of EL4-OVA-lymphoma-bearing C57BL/6 mice (day −6) treated with (*n*=7 per group) or without (*n*=4–5 per group) 5 × 10^6^ OT1 splenocytes (day −1) and additionally treated with or without LCMV (i.t. 2 × 10^6^ PFU, day 0). Data are shown as mean±s.e.m. and analysed by unpaired Student's *t*-test. NS, nonsignificant; *P<0.05, **P<0.01 and ***P<0.001.

So far, we found that monocytes and IFN-I were essential factors needed for tumour regression, and that T cells were dispensable for LCMV cancer therapy of early stage tumours. However, we wondered how the presence of CD8^+^ T cells might also contribute to tumour regression. We hypothesized that CD8^+^ T cells have a dual role in arenavirus-based therapy. On the one hand, they are essential in controlling virus, therefore limiting viral persistence. On the other hand, tumour-specific CD8^+^ T cells might be activated by arenavirus and might therefore contribute to tumour regression. Indeed, we found that virus-specific CD8^+^ T cells were induced during arenavirus therapy (Fig. 4a). The presence of virus-specific CD8^+^ T cells limited replication and effectiveness of LCMV therapy during systemic treatment (Supplementary Fig. 8a). Conversely, lack of virus-specific CD8^+^ T cells prolonged the antitumoural effects of IFN-I and enhanced survival even in an advanced tumour stage (Supplementary Fig. 8b,c).

To analyse the role of LCMV on tumour-specific CD8^+^ T cells, we infected mice bearing Ovalbumin-expressing B16F10 tumours in the presence of tumour-specific CD8^+^ T cells. LCMV enhanced expression of IL-2Rβ (CD122) and IL-7Rα (CD127) on tumour-specific CD8^+^ T cells, both of which promote cell survival (Fig. 4b)^8^. Moreover, LCMV infection enhanced tumour-specific CD8^+^ T cells in tumour infiltrates after vaccination with tumour antigen (Fig. 4c). Accordingly, the combination of LCMV and tumour-specific CD8^+^ T cells was most effective in suppressing tumour growth in the B16F10 melanoma model and in the EL4 subcutaneous lymphoma model (Fig. 4d,e). This suggests that LCMV enhances the infiltration and function of tumour-specific CD8^+^ T cells, which contributes to LCMV-mediated tumour regression.

### Reduced vasculature correlates with LCMV treatmen**t**

Next, we investigated the mechanism of IFN-I-induced tumour suppression in our models. As MOPC cells express the IFN-α/β receptor subunit 1 (IFNAR1) (Supplementary Fig. 2d), we analysed LCMV-induced tumour regression in *Ifnar*^*−/−*^ mice, which are deficient in the IFN-I receptor. LCMV infection equally suppressed tumour growth in wild type (WT) and *Ifnar*^−/−^ mice (Supplementary Fig. 9), suggesting that IFN-I secreted by infiltrating Ly6C^+^ monocytes directly acts on tumour cells. As IFN-Is are potent inhibitors of tumour associated angiogenesis^37^, we studied the expression of angiogenic factors by tumour cells in relation to LCMV treatment. Interestingly, almost all angiogenic regulators studied were suppressed by LCMV infection (Supplementary Fig. 10a). Moreover, CD31^+^ vessel formation was severely blunted in LCMV-infected tumours (Supplementary Fig. 10b), leading to reduced microvessel density (MVD) and increased vessel-to-vessel distances (Supplementary Fig. 10c). Consistent with reduced angiogenesis, we observed hypoxic areas in LCMV-treated tumours (Supplementary Fig. 10d) that were characterized by limited tumour cell proliferation and increased apoptosis (Supplementary Fig. 10e).

### Arenaviruses induce regression of human tumours

**Figure 5:**
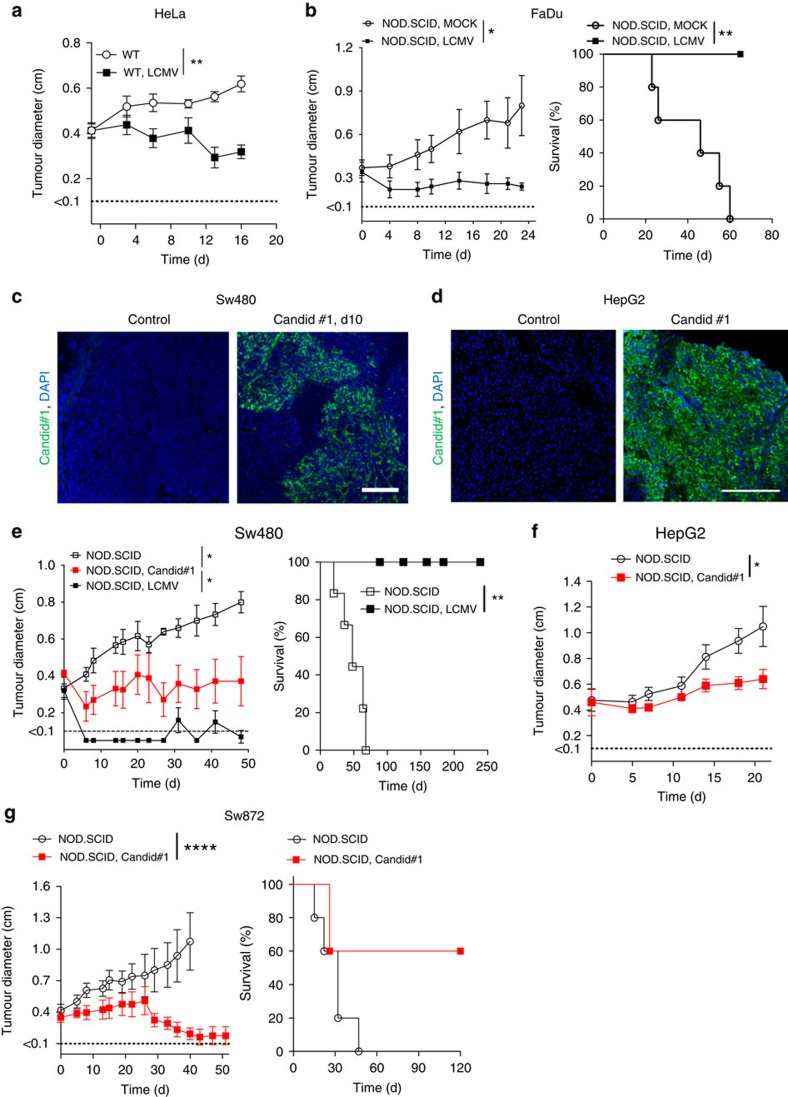
LCMV and arenavirus vaccine Candid#1 induce regression of human tumours. (**a**) Tumour diameter from HeLa-tumour-bearing NOD/SCID mice (day −8) treated with or without 2 × 10^4^ PFU LCMV intratumourally (*n*=8 per /group). (**b**) Tumour diameter and survival from FaDu-tumour-bearing NOD/SCID mice (day −10) treated with or without 2 × 10^6^ PFU LCMV i.t. (*n*=5 per group) (**c**) Immunofluorescence (day 10) of tumours from Sw480-tumour-bearing NOD/SCID mice (day −10) treated with or without 2 × 10^4^ PFU Candid#1 intratumoural (*n*=3 per group). Scale bar, 200 μm. (**d**) Immunofluorescence (day 10, *n*=3 per group) from HepG2-tumour-bearing NOD/SCID mice treated with or without 5 × 10^5^ PFU Candid#1 intratumourally. Scale bar, 200 μm. (**e**) Tumour diameter and survival from Sw480-tumour-bearing NOD/SCID mice (day −11) treated with (*n*=5) or without (*n*=6) 5 × 10^5^ PFU LCMV or 2 × 10^4^ PFU Candid#1 (*n*=7) intratumourally. (**f**) Tumour diameter from HepG2-tumour-bearing NOD/SCID mice treated with (*n*=5) or without (*n*=4) 5 × 10^5^ PFU Candid#1 intratumourally. (**g**) Tumour diameter (untreated *n*=6; treated *n*=8) and survival (*n*=5 per group) from Sw872 liposarcoma-bearing NOD/SCID mice treated with or without 5 × 10^5^ PFU Candid#1. Data are shown as mean±s.e.m. and analysed by unpaired Student's *t*-test. Survival is shown in Kaplan–Meier method and analysed by log-rank test. *P<0.05, **P<0.01 and ****P<0.0001

To explore whether arenavirus therapy could be translated to the human system, we used human xenograft models in NOD/SCID mice. Peritumoural LCMV injection led to regression of subcutaneously established HeLa and FaDu tumours in NOD/SCID mice (Fig. 5a,b). Next, we studied the clinically used Junin virus vaccine, Candid#1 (refs 11, 12). Candid#1 replicated in Sw480- and HepG2-tumours *in vivo* (Fig. 5c,d). Infection with LCMV and with Candid#1 limited growth of Sw480 tumours (Fig. 5e). Similarly, Candid#1 limited growth of HepG2 and Sw872 tumours (Fig. 5f,g). These data suggest that the vaccine virus Candid#1 has antitumoural properties much similar to the laboratory strain LCMV-WE.

### LCMV is superior to oncolytic viruses and PD1 blockade

**Figure 6:**
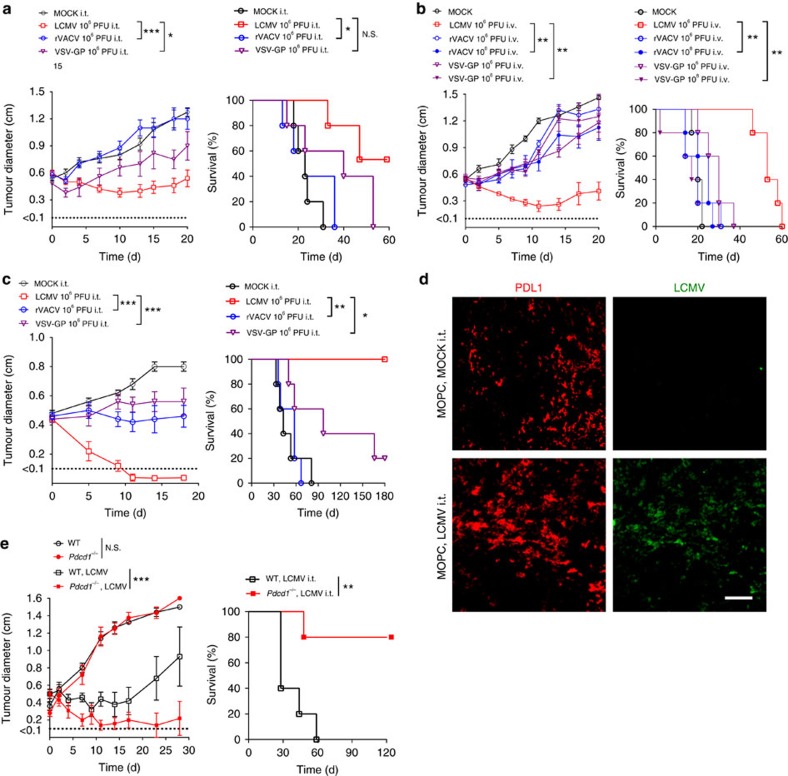
LCMV induces the strongest tumour regression compared with oncolytic viruses and PD1 blockade. (**a**,**b**) Tumour diameter and survival of MOPC-tumour-bearing C57BL/6 mice (day −10) treated intratumourally (**a**) or intravenously (**b**) with LCMV, VSV-GP, rVACV or vehicle (*n*=5 per group). (**c**) Tumour diameter and survival of Sw480-tumour-bearing NOD/SCID mice treated i.t. with 2 × 10^6^ PFU of LCMV, rVACV, VSV-GP or MOCK control (*n*=5 per group). (**d**) Immunofluorescence of tumours from MOPC-tumour-bearing mice (day −10) treated with or without 2 × 10^4^ PFU LCMV peritumourally on day 0 (LCMV, green; red PD-L1; *n*=3 per group). (**e**) Tumour diameter and survival of MOPC-tumour-bearing C57BL/6 and *Pdcd1*^*–/–*^ mice (day −10) treated with or without 2 × 10^4^ PFU LCMV peritumourally on day 0 (*n*=5 per group). Data are shown as mean±s.e.m. and analysed by unpaired Student's *t*-test. Survival is shown in Kaplan–Meier method and analysed by log-rank test. NS, nonsignificant; *P<0.05, **P<0.01 and ***P<0.001; scale bar, 200 μm.

Next, we compared LCMV with two oncolytic viruses presently in clinical or preclinical development: a chimeric variant of vesicular stomatitis virus (VSV-GP)^38^ and a recombinant TK-depleted vaccinia virus (TK^−^ VACV-GFP/LacZ, rVACV)^39^. Although VSV-GP is currently being developed (www.viratherapeutics.com), another very similar VSV variant (VSV-IFN) is being investigated in a Phase I study for hepatocellular carcinoma (ClinicalTrials.gov NCT01628640). rVACV is so far tested as a double deletion mutant (TK^−^ and VGF^−^) in a phase I study, with patients with advanced solid tumours^40^. In the MOPC tumour model, intravenous and intratumoural application of VSV-GP and rVACV showed limited antitumoural effects when compared with LCMV (Fig. 6a,b). Even a 100-fold higher dose of VSV-GP or rVACV did not reach the efficacy of LCMV-induced tumour regression in the MOPC tumour model (Fig. 6b). In line, human Sw480 xenografts were more susceptible to intratumoural LCMV therapy than to VSV-GP or rVACV (Fig. 6c). Next, we analysed the effects of PD-1 blockade together with LCMV treatment. We observed strong expression of PD-L1 in MOPC tumours (Fig. 6d). Blockade of PD-1 (in *Pdcd1*^*−/−*^ mice) had no impact on tumour regression in the MOPC model (Fig. 6e). In contrast, combination of PD-1 blockade with LCMV had the strongest impact on tumour regression in advanced tumour stage (Fig. 6e).

## Discussion

The ability of viruses to kill cancer cells has been recognized for several decades and is supported by recent Food and Drug Administration and European Union (EU) approval of an oncolytic herpesvirus (Talimogene laherparepvec, Amgen, Inc.)^41^. The specific mechanisms of virotherapy have long been considered to be directly oncolytic. Recently, it was recognized that induction of an inflammatory response contributes to virus-mediated tumour regression^42^,^43^,^44^,^45^,^46^,^47^,^48^,^49^. In cancer therapies with oncolytic viruses, this is considered to be a limited collateral effect. By contrast, in our study, we provide evidence that a powerful immune response itself induced by arenavirus replication may lead to complete tumour regression.

Treatment with a non-oncolytic arenavirus is advantageous in inducing sustained immune surveillance. First, an arenavirus such as LCMV will not kill the host cell by direct cytopathic effects. Therefore, virus replication is maintained until an immune response is induced within the tumour tissue. Second, arenavirus replication cannot solely be limited by a strong IFN-I response^9^,^50^,^51^. In addition, LCMV usually fails to induce rapid neutralizing antibodies^52^. Thus, arenavirus replication in tumours can only be controlled by infiltration of virus-specific CD8^+^ T cells. Consequently, as long as CD8^+^ T cells do not infiltrate the tumour, arenaviruses can replicate for several days or weeks even if tumour cells respond to IFN-I. In a direct comparison of the IFN-I-responsive tumour models MOPC and Sw480, we found that LCMV was therapeutically more potent than the oncolytic viruses VSV-GP and rVACV. These results could have been expected, because, in contrast to LCMV, VSV-GP and rVACV require defects in the IFN pathway for efficient replication in tumour cells^53^,^54^,^55^. Consequently, arenavirus therapy could fill a gap in virotherapy in the treatment of IFN-I-responsive cancers. Careful characterization of IFN-I responsiveness and additional LCMV host factors may guide the selection of patients suitable for LCMV cancer therapy.

Treatment with arenaviruses might also have certain disadvantages. The broad tropism of arenaviruses^56^ might induce adverse events. Live arenaviruses could also replicate in healthy, non-malignant tissue. This replication could induce an immune response in normal tissue, leading to immunopathological side effects^9^,^52^. Although >5% of humans show evidence of previous LCMV exposure without severe symptoms^10^, especially in highly immunocompromised patients overwhelming replication of attenuated vaccine virus could lead to life-threatening disease^52^,^57^ or to gain of selection mutants of the virus^58^. Both risks can be limited by intervention with neutralizing antibodies against the arenavirus strain or antiviral therapy^57^. In our murine models we observed initially enhanced LCMV replication in the presence of tumours, but viral replication lead to an immune response that was capable to control intratumoural virus load. Using the hepatotropic LCMV strain WE, we observed some viral replication in livers of B16F10 mice, but liver enzymes were under the upper limit of normal when tested 20 days after virus injection. LCMV-treated WT, transgenic and NOD/SCID mice did not show any virus or immunopathology related signs of sickness. Nevertheless, the clinical applicability of arenavirus-based treatment has to be carefully evaluated.

We found that arenavirus-based tumour therapy required IFN-I. IFN-I has substantial antiproliferative activity in different cancer types^59^. Three major mechanisms could be responsible for these effects: first, IFN-I can directly induce cell cycle arrest in cancer cells^60^,^61^; second, IFN-I can trigger an antigen-specific antitumour immune response^4^; and third, IFN-I can inhibit angiogenesis^37^. We found in the MOPC model that arenavirus infection enhanced IFN-I induction, enhanced antitumour immune response and limited angiogenesis.

LCMV treatment did not influence PD-L1 expression in the tumour nor PD-1 expression on CD8^+^ T cells. In contrast, it enhanced tumour-infiltrating tumour-specific CD8^+^ T cells, as well as survival signals in these cells. In line with these findings, a combination of CD8^+^ T cells and LCMV, or a combination of PD-1 blockade and LCMV, were the most effective tumour treatments.

To date, different strains of LCMV are in laboratory use for the investigation of acute and chronic viral infections, as well as an efficient tool to enhance and examine T-cell responses in murine models of infection, autoimmune disease and cancer^13^,^15^,^56^,^62^. For example, Schadler *et al*.^14^ used the low replicative ‘neurotropic strain' LCMV Armstrong (LCMVarm) in addition to lipopolysaccharide (LPS) and anti-CD3, to increase thrombospondin-1 (Tsp1) in T cells and tested the antitumoural potency of Tsp1. In their study, growth of B16F10 melanomas was observed for approximately 2 weeks after LCMVarm infection and antitumoural effects after LCMVarm administration were dependent on Tsp1 and T cells. By contrast, our studies with the ‘viscerotropic strain' LCMV-WE revealed that its potent antitumour activity was mediated via IFN-I and was T-cell independent. The differences in mechanism and potency underlying the antitumour effects of LCMV in the two studies may reflect strain-dependent differences between the different arenaviruses used, the impact of the specific cancer types examined and the different timepoints used. Thus, similar to Schadler *et al*.^14^ we observed an impaired antitumoural effect of LCMV in *Rag1*^*−/−*^ mice compared with WT mice within the first 2 weeks after LCMV treatment of established tumours, indicating the involvement of T cells (Supplementary Fig. 8b, middle panel). However, in the long-term observation of approximately 80 days, we show that the absence of T cells is beneficial for LCMV-WE therapy (Supplementary Fig. 8b). Moreover, we based our studies largely on the mechanism of antitumoural action of LCMV in a murine head and neck cancer model (MOPC), whereas Schadler *et al*.^14^ used malignant melanoma, where effective immunotherapies mainly depend on tumour-specific T cells^63^. This may also explain the lower efficiency and efficacy of IFN-dependent LCMV therapy on B16F10 (Fig. 2f) compared with MOPC (Fig. 2a) or MC38 colon cancer (Fig. 2e).

Studies from others, such as Ochsenbein *et al*.^13^ and Flatz *et al*.^15^, used LCMV as a model to activate tumour-specific CD8^+^ T cells. In agreement with their work, we use transgenic tumour models to show that OVA-specific T cells show enhanced antitumoural effects, when additional T-cell activation via LCMV infection is performed. However, we demonstrate that T cells exhibit an ambiguous role in LCMV treatment of tumours. In the presence of tumour-specific T cells, LCMV enhances antitumoural immune defenses, but the presence of virus-specific T cells limits the antitumoural activity of LCMV by controlling viral replication (Supplementary Fig. 4). Moreover, studying the mechanism of antitumoural action of LCMV-WE, we found that T cells were largely dispensable in our MOPC tumour model in *Tcrab*^*−/−*^, *Rag1*^*−/−*^ and NOD/SCID mice. Rather, LCMV-induced antitumour immunity depends mainly on the recruitment of IFN-producing Ly6C^+^ monocytes.

We found that monocytes, which infiltrate into human tumours, have the ability to produce IFN-I. It remains an open question whether local or systemic injection of Candid#1 in humans could be similar to mice in enhancing the local IFN-I response and thereby inducing tumour regression. In recent times it was found that IFN-free therapy of chronic hepatitis C virus-related hepatocellular cancer leads to unexpected early tumour recurrence^64^. This finding underlines the potent antitumoural effects of IFN-I on cancer growth during persistence of virus.

In conclusion, we found that locally or systemically administered arenaviruses preferentially replicate in murine and human cancer cells, and strongly induce a local innate immune response, leading to effective regression of disseminated cancers *in vivo*.

## Methods

### Human material

Formalin-fixed, paraffin-embedded primary tumour tissue was retrieved from the archives of the Department of Otorhinolaryngology, University Hospital Essen, from patients who had signed their informed consent according to the Declaration of Helsinki and with approval from the institutional review board at the University Hospital Essen.

### Viruses

The LCMV strain WE and VSV (strain Indiana) were kindly provided by Rolf Zinkernagel (Institute of Experimental Immunology, ETH, Zurich, Switzerland). LCMV was propagated in L929 cells, which were purchased from ATCC (CCL-1). VSV was propagated in BHK cells, which were bought from ATCC (CRL-8544). Virus titres in tissue of infected mice were measured using plaque assays. For detection of virus in the skin, about 10 mg of the infected skin area was used. Candid#1 was grown in Vero E6 cells purchased from ATCC (CRL-1586). Recombinant VSV-GP (provided by Professor von Laer, Division of Virology, Medical University Innsbruck, Austria) was generated as previously described^38^. VSV-GP was grown on Vero cells under serum-free conditions. Recombinant Tk− Vaccinia virus strain Western Reserve containing the green fluorescent protein gene expressed under the P7.5 promoter within the Tk (thymidine kinase) locus of the genome (originally provided by B. Moss, National Institutes of Health, Bethesda, MD) was propagated, purified and titrated following standard methodology^65^.

### Cell lines and depletion antibodies

MOPC cells (murine oropharynx cancer) were initially named MTEC and were provided by Dr H.J. Lee of the University of Iowa. Cells were cultured in previously described culture medium^17^. MaMel66a cells (human malignant melanoma) were provided by Professor Schadendorf^66^.

MC38-OVA (MC38 cells, murine colon carcinoma) and EL4-OVA cells (murine ovalbumin-expressing lymphoma) were a kind gift of Bertrand Huard (University Medical Center, Geneva, Switzerland). B16F10 cells (murine malignant melanoma) were purchased from ATCC (CRL-6475). B16-OVA (OVA-expressing B16 malignant melanoma cells) were provided by Professor P. Knolle, Technical University, Munich, Germany. SW480 (human colon carcinoma, CCL-228) and FaDu cells (human oropharyngeal carcinoma, HTB-43), A549 cells (human bronchial carcinoma, CCL-185), HepG2 cells (human hepatoma, HB-8065) and HeLa cells (human cervix carcinoma, CCL-2) were purchased from ATCC. SW872 cells were kindly provided from Professor Bauer (University Hospital, Essen, Germany).

Human normal untransformed cells were purchased from ATCC (FHC CRL-183), LONZA (NHBE CC-2540; HRE CC2556) and PromoCell (NHEM C-12453), which were cultured and maintained according to the companies' protocols.

Monoclonal fluorescence-labelled antibodies against LCMV-NP (VL4) and LCMV-GP (clone KL25), and unlabelled anti-NK1.1 antibodies were produced in-house (Professor P.A. Lang, Heinrich Heine University, Düsseldorf, Germany).

Anti-Ly-6G (1A8), anti-Gr-1 Ab (RB6-8C5) and anti-IFNAR1 (clone MAR1-5A3) depletion antibodies were purchased from Bio X Cell. All other antibodies used for immunofluorescence are listed in Supplementary Table 1.

### Mice

C57BL/6J (Jackson Laboratory; 00664), *Map3k14*^*aly/aly*^ (Professor Shibata, Kyoto, Japan), *Ifnar1*^*−/−*^ (Jackson Laboratory; 028288), *Tcrab*^*−/−*^ (Jackson Laboratory; 002116), *Jh*^*−/−*^ (Jackson Laboratory; 002438), *Rag1*^*−/−*^ (provided by Professor P.A. Lang, Heinrich Heine University, Düsseldorf, Germany), *Ccr2*^*−/−*^ (Jackson Laboratory; 004999), OT-1 (kindly provided by Professor J. Fandrey, University Hospital, Essen) and *Irf3 x Irf7*^*−/−*^ (Provided by Professor Löhning, Charite Hospital Berlin) were maintained on the C57BL/6 background. IFNβ^mob/mob^ mice (provided by Professor Scheu, University of Düsseldorf, Germany) express the yellow fluorescent protein under the IFNβ promoter and therefore IFN-I-producing cells can be detected in fluorescein isothiocyanate channel^30^. The spontaneous tumour models LoxP-TAg9 (liver cancer) and MT*/ret* (melanoma) were used on C57BL/6 background. LoxP-TAg9 mice were provided by Professor Willimsky (Charite, University of Berlin, Germany). MT*/ret* mice were provided by Dr Helfrich (University Hospital Essen, Germany). For human xenografts, NOD/SCID mice were used (Charles River; 001303). Six- to 8-week-old, age- and sex-matched mice were used for all the studies. All mice were maintained in single ventilated cages, with the authorization of the Veterinäramt Nordrhein Westfalen (Düsseldorf, Germany) in accordance with the German law for animal protection or institutional guidelines of the Ontario Cancer Institute.

For *in vivo* depletion of NK cells, 50 μg anti-NK1.1 (in 400 μl, in house) was injected intraperitoneally (i.p.) on days −3 and −1. For depletion of Ly-6C and Ly-6G cells (monocytes and granulocytes), 200 μg anti-Ly6C+G antibody (Gr-1, clone RB6-8C5, Bio X Cell BE0075) was given i.p. on days −2, 2 and 7. For depletion of Ly6-G cells (granulocytes), 500 μg anti-Ly6G (clone 1A8, Bio X Cell BE0075-1) was given starting on day −2 and repeated on day 2 and day 7. For depletion of IFNAR1, 1 mg anti-IFNAR1 antibody (clone MAR1-5A3, Bio X Cell BE0241) was given i.p. on day −1 and repeated by i.p. injections of 250 μg IFNAR1 on days 2 and 8. Same amounts of the IgG1 Isotype control (clone MOPC21, Bio X cell) were injected to the control group. PolyI:C was provided by Sigma (P9582).

### Therapeutic application of arenavirus

The time of infection with virus was set to day=0. In subcutaneous tumour models, tumour cells where injected 13–3 days before as stated (that is, day −10). The optimal dose to guarantee persistence of virus in the tumour was 2 × 10^4^ PFU LCMV peritumourally or 2 × 10^6^ PFU intravenously. Viral doses are indicated in the figure legends. Cell supernatant was used for vehicle control.

### Histological analysis and immunocytochemistry

Histological analysis were performed on snap-frozen tissue as previously described^67^. In brief, sections were fixed with acetone for 10 min and nonspecific antigens were blocked in PBS containing 2% FCS for 15 min, followed by various stainings for 45 min.

Immunocytochemistry was performed on cells grown on coverslips^50^. In brief, cells were fixed in 4% Formalin/PBS for 10 min, washed in PBS and then permeabilized in 1% Triton X–PBS for 10 min. After blocking with 10% FCS/PBS for 30–60 min, various stainings for 45 min were followed. Coverslips were mounted on microscope slides using mounting medium (S3023, Dako).

Images were acquired with a fluorescence microscope (KEYENCE BZ II analyser). Quantifications were performed using the Image J software (NIH).

### Morphometric analysis of tumour vessels and hypoxia

Morphogenic analyses were performed using consecutive cryosections stained for the endothelial cell marker CD31. MVD was quantified by using the average of three tumour sections per tumour (top, middle and base). For MVD and vessel–vessel distances *n*=1,168 peritumoural vessel–vessel distances (50–100 vessels/tumour), 5 regions of interests per sample were quantified from *n*=3 histological samples. MVD was calculated as number of vessels per tumour area. Vessel-to-vessel distance was calculated using the middle section of the three generated using corresponding Cell P Software (Olympus, Germany). For analysis of hypoxic tumour areas, Pimonidazole was injected 30 min before killing the mice. Hypoxic tumour areas were detected by the formation of pimonidazole adducts. Tumour sections were immunostained using the Hypoxyprobe-1 Plus kit according to the manufacturer's protocol (Natural Pharmacia International, Inc.) and the hypoxic area index quantified as the percentage of positive tumour area per total tumour area in tumours of corresponding volumes.

### Flow cytometric analysis

Single-cell suspensions of cells were incubated with anti-CD16/32 (anti-FcgIII/II receptor, clone 2.4G2) in a 1:100 dilution for 10 min, then stained with conjugated antibodies. All fluorescently labelled monoclonal antibodies (Supplementary Table 1) were diluted 1:100 to their original concentration in FACS buffer. Tetramer was used from NIH tetramer core facility. All stained cells were analysed on LSRII/FACS Fortessa (BD Biosciences) flow cytometer and data were analysed with Flowjo software.

### IFN-α ELISA assay

Serum IFN-α levels were determined according to the manufacturers' specifications (Research Diagnostics RDI, Flanders, NJ).

### Reverse transcription and qRT–PCR

Total RNA was isolated by using TRIzol (Ambion), reverse transcribed into complementary DNA using Quantitect Reverse Transcription Kit (Qiagen) and analysed by qRT–PCR using the SYBR Green Master Mix (Applied Biosystems, Darmstadt, Germany) or using Taqman gene expression assay (Life Technologies). Either commercially available Primer sets (Supplementary Table 2–4) or self-designed primers ordered from Eurofins Genomics (Ebersberg, Germany) (Supplementary Table 5) were used. For analysis, the expression levels of all target genes were normalized to either 18S or glyceraldehyde 3-phosphate dehydrogenase messenger RNA expression. Relative gene expression levels were calculated with the ΔCt method.

### VSV assay

Cells per well (1 × 10^5^) were plated in a 24-well plate on day −1. On day 0, cells were treated with different concentrations of human IFNα4 (11100-1, PBL Assay Science), murine IFNα4 (12115-1, PBL Assay Science) or murine IFNα2 (14-8312-62, eBiosciences) and then infected with 500 PFU/ per well VSV. After 2 h Methylcellulose overlay was added. On day 1, cell layer was stained with crystal violet.

### Small interfering RNA transient transfections

MCF7 cells were seeded at 4 × 10^4^ cells per well in a 24-well plate and, 24 h later, transfected with control or B-Myb small interfering RNA duplexes (Origene SR419327) using Hiperfect (Qiagen 301705) as a transfection reagent. Transfected cells were infected for 24 h with LCMV-WE, 24 h post-small interfering RNA transfection.

### Statistical analysis

Mean values were compared using an unpaired Student's two-tailed *t*-test. Data are expressed as means±s.e.m. Student's *t*-test was used to detect significant differences between groups. Chi-Quadrat test was additionally used. Survival was compared with log-rank (Mantel–Cox) tests. The level of statistical significance was set at *P*<0.05.

### Gene set enrichment analysis

RNA-sequencing gene expression data from the TCGA for different types of cancer were downloaded using the cgdsr package of the cBioportal gateway for Cancer Genomics (http://www.cbioportal.org) and mRNA expression z-scores (RNA Seq V2 RSEM) used. TCGA tumours were ranked based on CD14 expression and selected top and bottom samples used as the input for GSEA. GSEA was performed with 1000 permutations using the Browne IFN-response gene set.

### Data availability

The authors declare that all data supporting the findings of this study are available within the article and its Supplementary Information files, or are available from the authors upon request.

## Additional information

**How to cite this article:** Kalkavan, H. *et al*. Spatiotemporally restricted arenavirus replication induces immune surveillance and type I interferon-dependent tumour regression. *Nat. Commun.* **8,** 14447 doi: 10.1038/ncomms14447 (2017).

**Publisher's note**: Springer Nature remains neutral with regard to jurisdictional claims in published maps and institutional affiliations.

## Supplementary Material

Supplementary Information Supplementary Figures and Supplementary Tables
