# Supplementary material for: Spatiotemporally restricted arenavirus replication induces immune surveillance and type I interferon-dependent tumour regression
Source: Nat Commun. 2017 Mar 1;8:14447. doi: 10.1038/ncomms14447 (PMC5337983; doi:10.1038/ncomms14447)
Supplement: Supplementary Information — Supplementary Figures and Supplementary Tables [file ncomms14447-s1.pdf]

Supplementary Figure 1

a

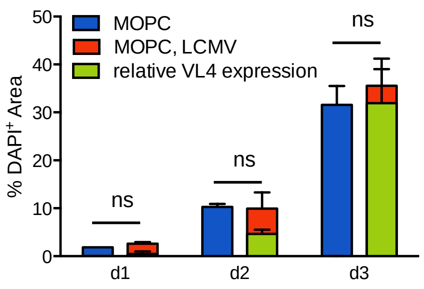

b

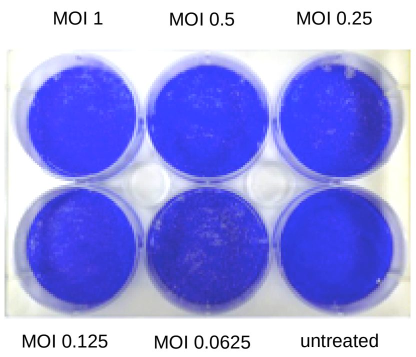

Supplementary Figure 1: LCMV replicates in MOPC without affecting cell proliferation
a: Quantification of Immunofluorescence of MOPC cells untreated or infected with LCMV (MOI 1) 72 hours after infection (n = 3/group) (related to Fig. 1c). ns, non-significant. **b:** Non-impaired clonogenic survival of MOPC cells after in vitro LCMV WE infection. Representative photograph of long-term colony formation following incubation of MOPC with LCMV WE of indicated MOI.

Supplementary Figure 2

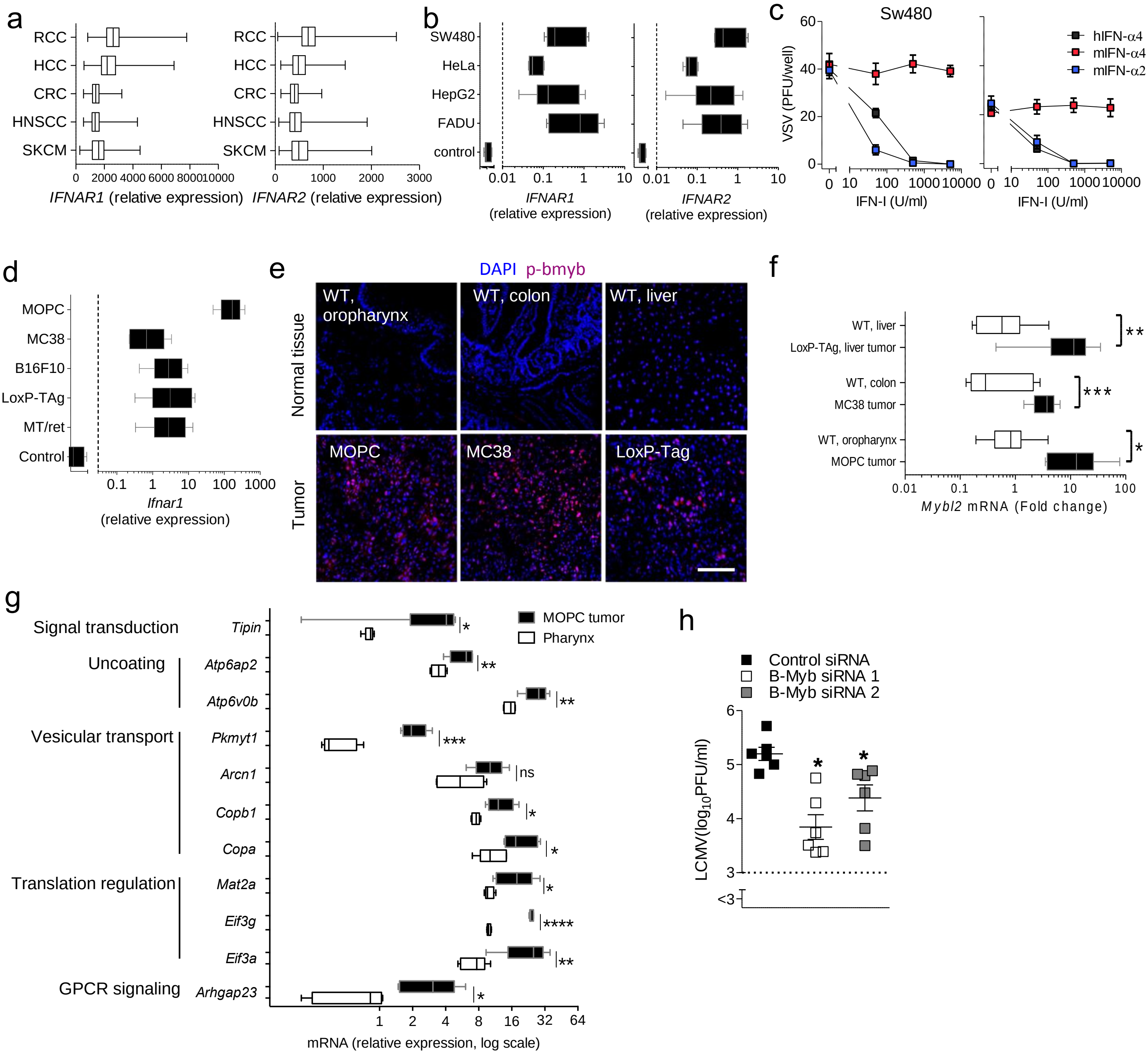

Supplementary Figure 2: B-Myb expression, and not IFN-I unresponsiveness, is one factor promoting arenavirus replication

a: mRNA expression levels of *Ifnar1* and *Ifnar2* for the cancer types renal cell cancer (RCC; n=534), hepatocellular cancer (HCC; n=373), colorectal cancer (CRC; n=382), head and neck squamous cell cancer (HNSCC; n=522) and skin cutaneous melanomas (SKCM; n=471). Downloaded from the TCGA database by use of cBioPortal (www.cbioportal.org). Data are shown as mean and minimum-maximum whiskers. **b:** qRT-PCR of human *Ifnar1* and *Ifnar2* mRNA expression in Sw480, HeLa, HepG2, FADU cells and controls (murine mRNA, n=5/group). **c:** Number of viral plaques in Sw480 cell layer (n=6/group, left panel) and HeLa cell layer (n=5/group, right panel), which were treated with different concentrations of human IFNα4, murine IFNα4 or murine IFNα2 and infected with VSV (MOI 0.01) 24 hours earlier. **d:** qRT-PCR of murine *Ifnar1* mRNA in MOPC cells, MC38 cells, B16F10 cells, LoxP-Tag tumors, MT/ret cells and control (human mRNA, n=5/group). **e&f:** Immunofluorescence of phospho-B-Myb (e, n=3/group) and qRT-PCR of B-Myb (=*Mybl2*) mRNA (f, n = 9-10/group) in MOPC, MC38 and LoxP-Tag tumors compared to the correlating healthy tissue (oropharyngeal epithelia, colon and liver). Scale bar, 200µm. **g:** qRT-PCR of viral host factors in MOPC tumors (n=5) and pharynx tissue (n=5). **h:** Infectious LCMV particles in supernatant of MCF7 cells, which were treated with control siRNA or two separate B-Myb siRNAs and then 24 hours later infected with LCMV (MOI 1) analyzed 24 hours after infection (n = 6/group). Data are shown as mean ± SEM and analyzed by unpaired Student`s t-test. ns, non significant; *p < 0.05; **p < 0.01; ***p < 0.001.

Supplementary Figure 3

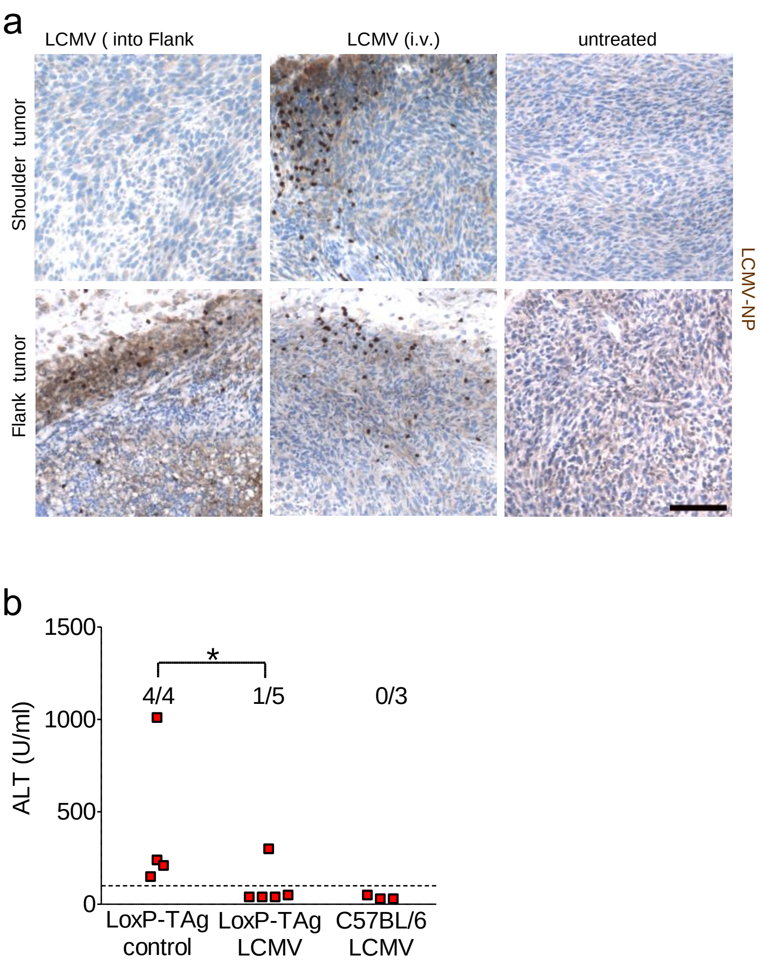

Supplementary Figure 3: Intravenous infection leads to viral replication in metastasis
a: Immunohistochemistry for LCMV-NP in flank and shoulder tumor (day 15) from C57BL/6 mice receiving simultaneously subcutaneously 5 x 10^5 MOPC cells in the flank and shoulder (day -3), treated with or without $2x10^4$ PFU LCMV given into the flank or intravenously on day 0 (n=4-5/group). Scale bar, 200µm. **b:** Serum alanin-aminotransferase (ALT) levels of C57BL/6 (n=3) and adenovirally induced tumor bearing LoxP-TAg mice (11 month old) which were left untreated (n=4) or which were additionally treated with 2 x 10^6 PFU of LCMV-WE (n=5) measured 20 days after infection. Values above line indicate pathological liver disease.

Supplementary Figure 4

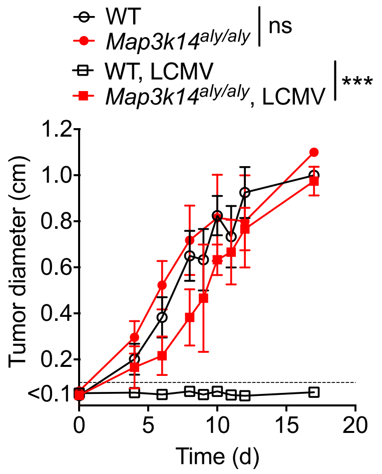

Supplementary Figure 4: Immune cell infiltrates are required for tumor regression
Tumor diameter of MOPC-tumor bearing WT and *Map3k14*$^{aly/aly}$ mice (day -3) treated with (n=8 WT; n=6 *Map3k14*$^{aly/aly}$) or without (n=7 WT; n=6 *Map3k14*$^{aly/aly}$) 2x10^4 PFU LCMV peritumorally on day 0. Data are shown as mean ± SEM and analyzed by unpaired Student`s t-test. ns, non significant; ***$p < 0.001$.

Supplementary Figure 5

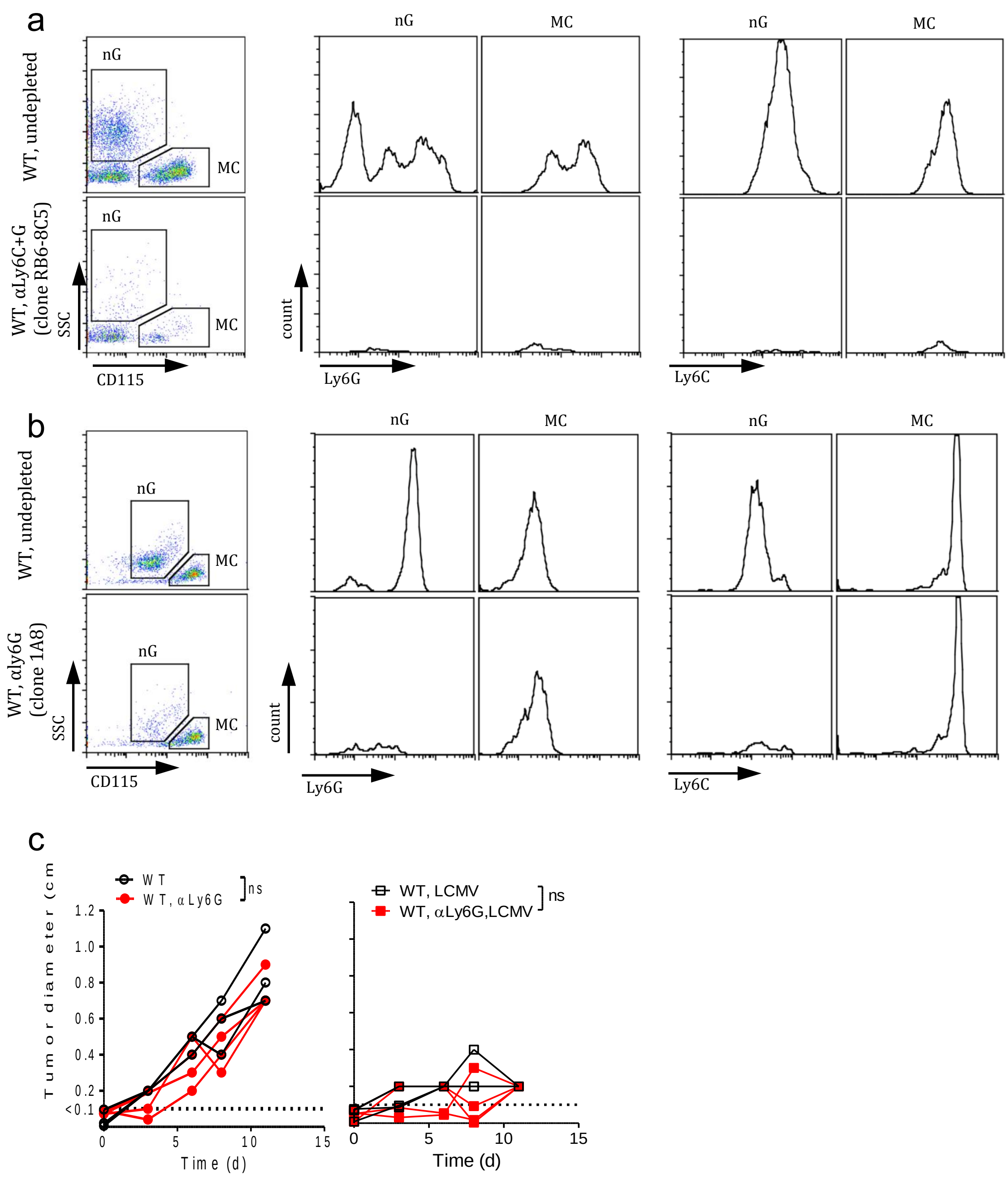

Supplementary Figure 5: Tumor infiltrating Ly6C+ cells are indispensable for antitumoral LCMV therapy

MOPC tumor bearing C57BL/6J mice were treated with $2x10^4$ PFU LCMV peritumorally on day 0. Depletion was performed by i.p. injection of either (a) αLy6C+G (clone RB6-8C5) 200µg or (b and c) αly6G (clone 1A8) 500µg starting on d-2 and repeated on d2 and d7. **a&b:** Representative dot blots of peripheral vein blood collected on d4 and stained for monocyte and granulocyte cell markers after preselection of CD11b positive cells. **c:** Tumor diameter in C57BL/6 mice injected with or without αLy6G (1A8) antibody (n=3-4/group).

Data are shown as mean ± SEM and analyzed by unpaired Student`s t-test. ns, non-significant.

Supplementary Figure 6

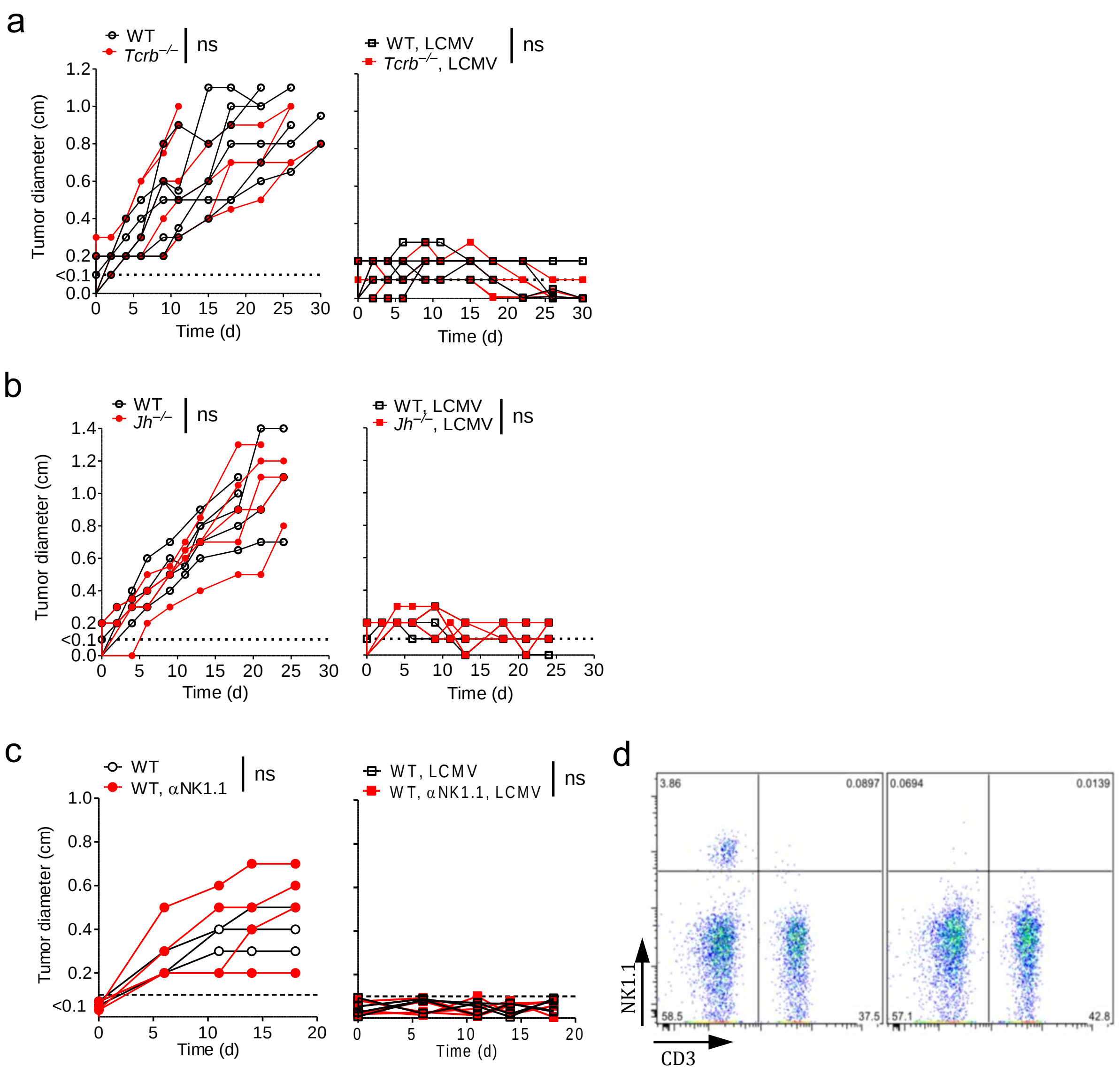

Supplementary Figure 6: Impact of adaptive immune system and NK cells on LCMV-induced anti-tumoral immunity.

a: Tumor diameter of MOPC-tumor bearing WT and *Tcrab*$^{-/-}$ mice (day -3) treated with or without 2x10^{4} PFU LCMV peritumorally on day 0 (n=5/group). **b:** Tumor diameter of MOPC-tumor bearing WT and *Jh*$^{-/-}$ mice (day -3) treated with or without 2x10^{4} PFU LCMV peritumorally on day 0 (n=5/group). **c&d:** Tumor diameter (c) and representative dot blot from peripherial vein blood collected at day 2 and stained for NK cell markers (d) of WT mice which were NK depleted by i.p. injection of 400µl NK1.1 antibody on day -3 and day -1 (n = 4/group).

Data are shown as mean ± SEM and analyzed by unpaired Student`s t-test. ns, non-significant.

Supplementary Figure 7

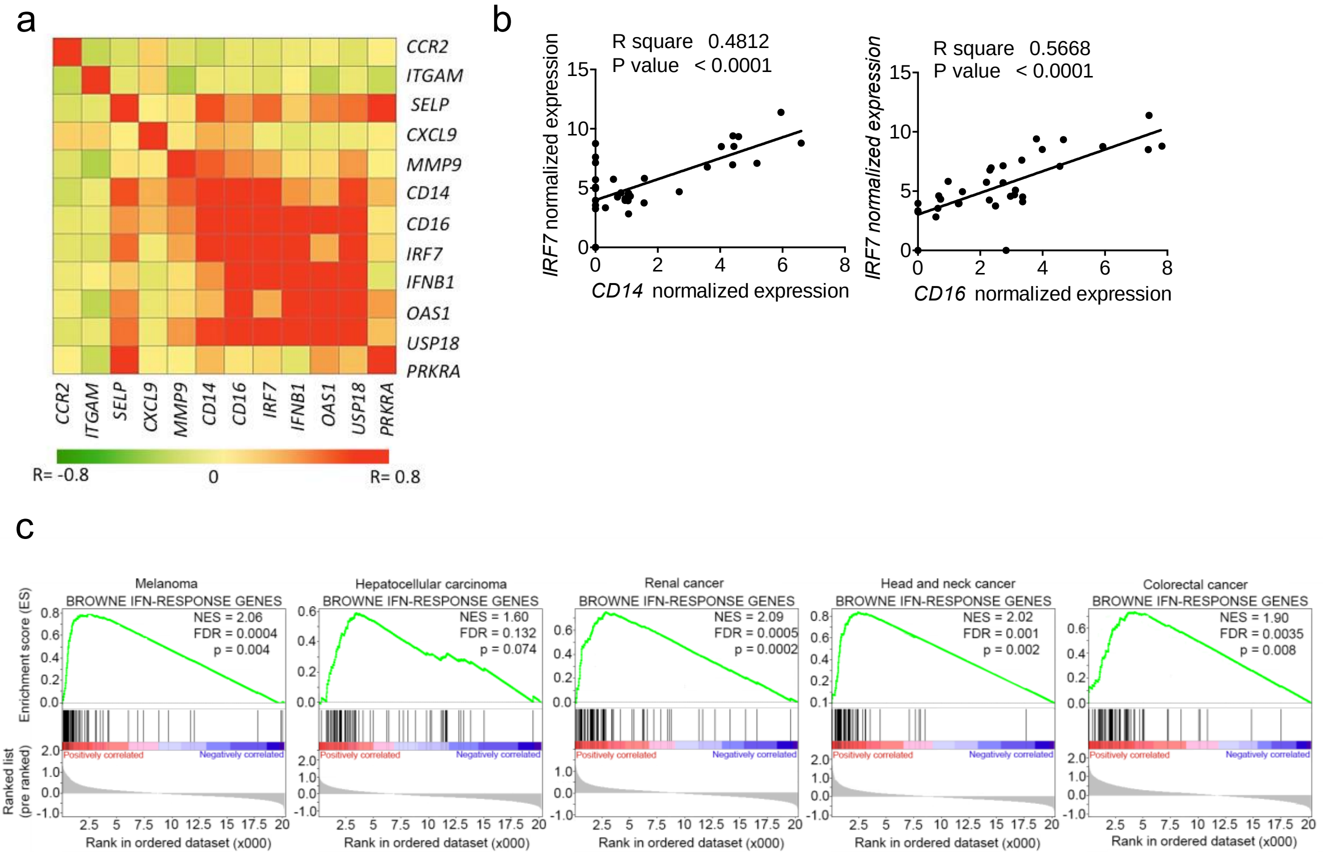

Supplementary Figure 7: Monocyte recruitment in human cancers correlates with IFN-I induction

a: Heatmap of correlation analyses of 12 immunogenes mRNAs of primary samples from 34 oropharyngeal cancer patients. **b:** Linear regression between *IRF7* and the human monocyte markers *CD14* and *CD16*. **c:** Gene-set enrichment analysis (GSEA) of human IFN-I induced genes by usage of the Browne interferon-related gene-set. IFN-I-induced expression profile and genes ranked by extent of differential expression in TCGA melanoma, hepatocellular carcinoma, renal cancer, head and neck cancer and colorectal cancer dataset. NES, normalized enrichment score; FDR, false discovery rate.

Supplementary Figure 8

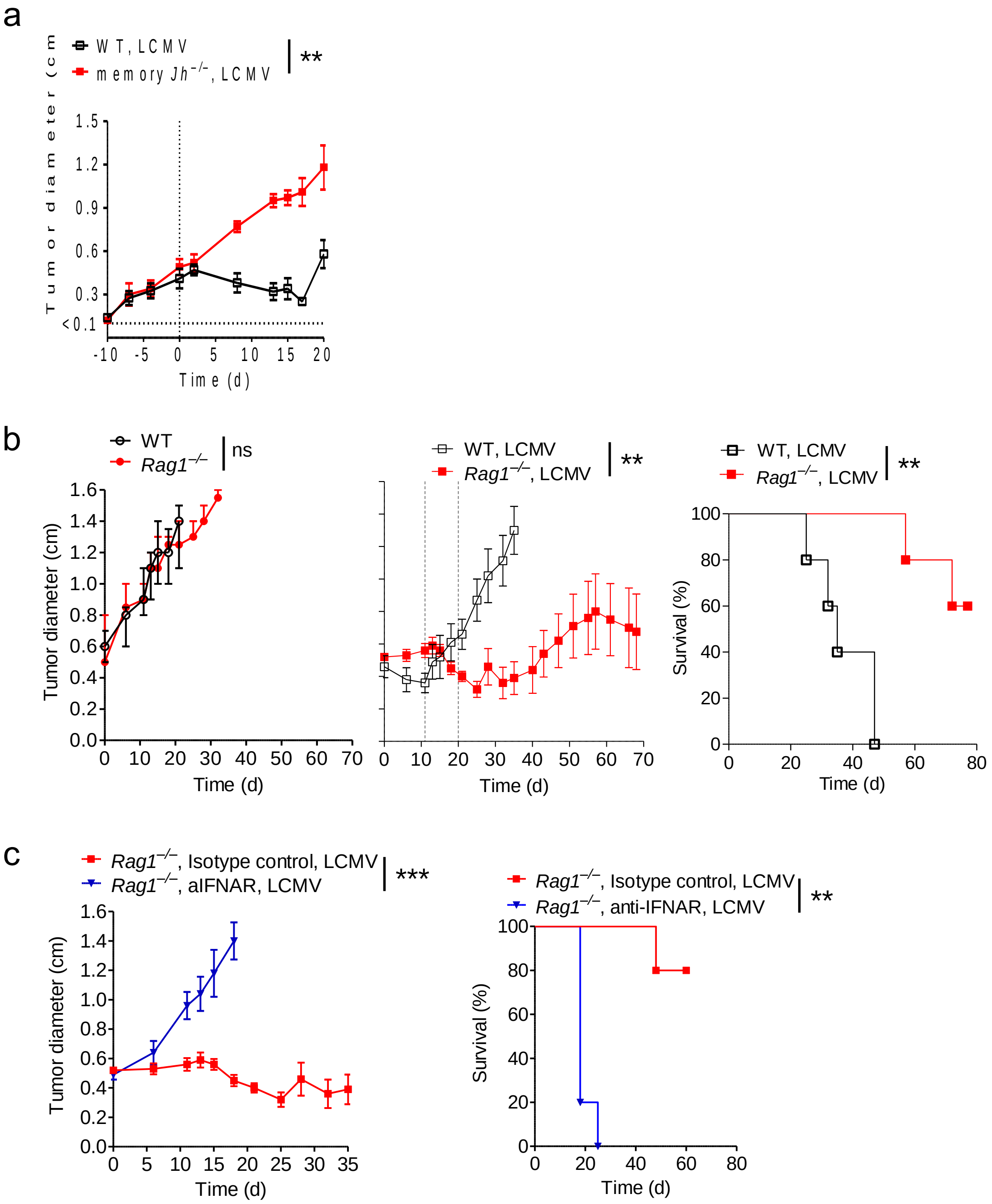

Supplementary Figure 8: Virus-specific memory $CD8^+$ T cells prevent LCMV-induced tumor regression

a: Tumor diameter of MOPC-tumor bearing WT or memory *Jh*$^{-/-}$ mice (pretreated with 200 PFU LCMV i.v. 100 days before tumor inoculation) treated with LCMV $2x10^4$ PFU intravenously on day 0 (n = 5/group). **b:** Tumor diameter and survival from MOPC-tumor transplanted WT and *Rag1*$^{-/-}$ mice (day -10) treated with or without $2x10^4$ PFU LCMV intratumorally on day 0 (n = 5/group) **c:** Tumor diameter and survival from MOPC-tumor transplanted *Rag1*$^{-/-}$ mice (day -10) treated with anti-IFNAR1 antibody or isotype control (n = 5/group). Data are shown as mean ± SEM and analyzed by unpaired Student`s t-test. Survival is shown in Kaplan-Meier method and analyzed by log-rank test. ns, non-significant; *p < 0.05; **p < 0.01; ***p < 0.001.

Supplementary Figure 9

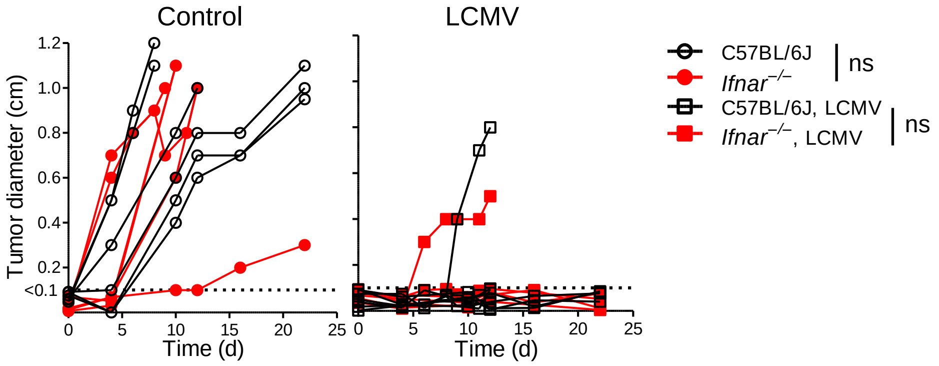

Supplementary Figure 9: Anti-tumoral effect of LCMV is independent of host IFNAR expression. Tumor diameter of *Ifnar*$^{-/-}$ and C57BL/6 control mice injected with 5x10^{5} MOPC cells (day -3) subcutaneously and either left untreated (left) or treated with 2x10^{4} PFU LCMV peritumorally (right, day 0) (n = 6-7/group). Measurements were performed on the indicated days. ns, non-significant.

Supplementary Figure 10

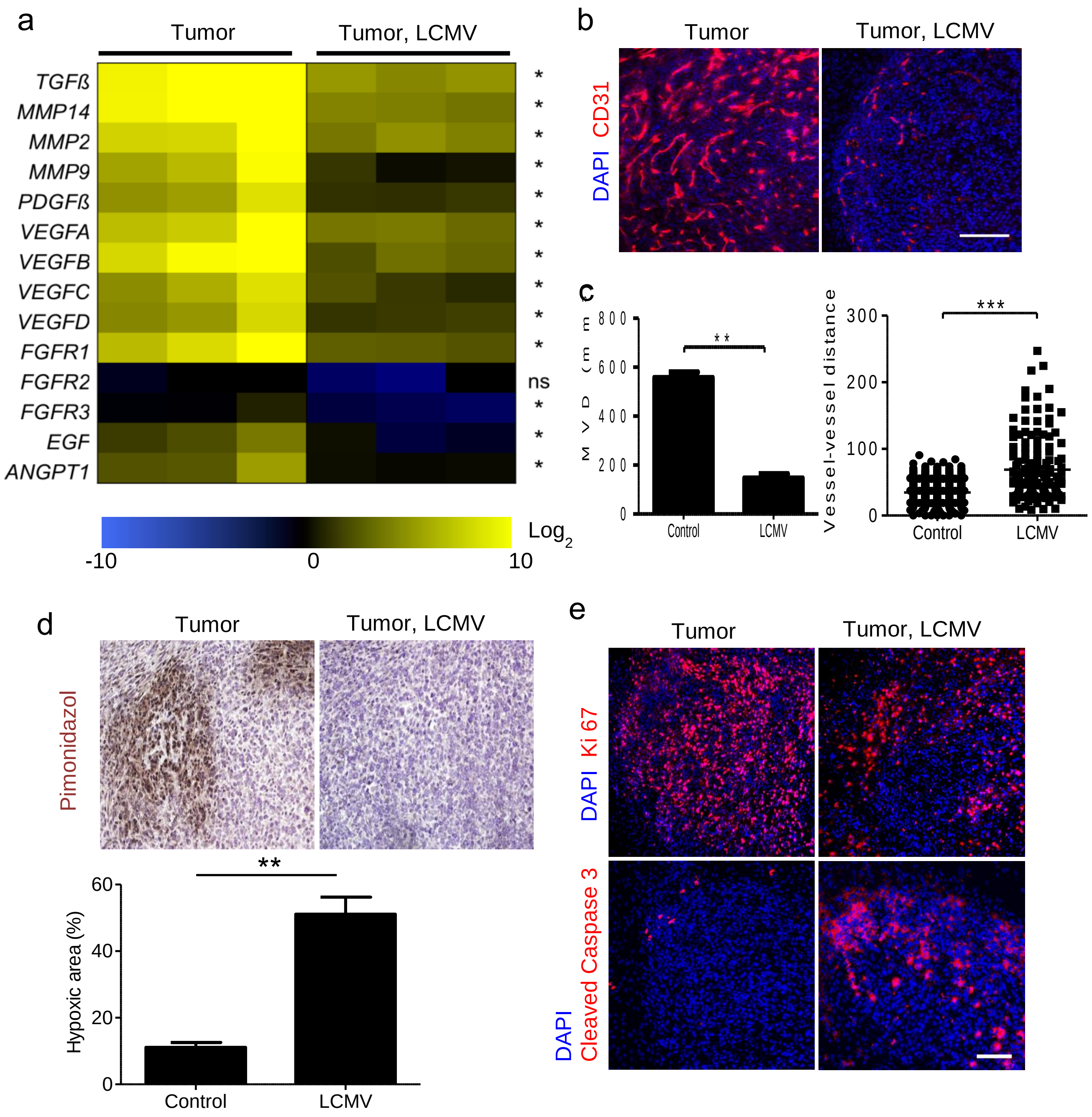

Supplementary Figure 10: Reduced vascularisation correlates with arenavirus mediated tumor regression

a: qRT-PCR from tumors of MOPC-tumor bearing WT mice (day -3) treated with or without 2x10^4 PFU LCMV peritumorally (day 0), analyzed on day 6 (n= 3/group). **b&c:** Immunofluorescence (b, n= 3/group, CD31, red; DAPI, blue) and quantification of microvessel density (MVD) and vessel–vessel distances (c, n = 3/group) from MOPC-tumor bearing C57BL/6 mice (day -3) which were treated with or without 2x10^4 PFU LCMV peritumorally (day 0) measured on day 6. **d:** Immunohistochemistry (day 6) and quantification (day 9) of hypoxic areas from tumors of MOPC-tumor bearing C57BL/6 mice (day -3) treated with or without 2x10^4 PFU LCMV peritumorally on day 0 (n= 3 mice/group). **e:** Immunofluorescence of tumors from MOPC-tumor bearing C57BL/6 (day -3) treated with or without 2x10^4 PFU LCMV peritumorally on day 0 (n= 3/group, Ki-67 or cleaved caspase 3, red; DAPI, blue). Data are shown as mean ± SEM and analyzed by unpaired Student`s t-test. ns, non-significant; *p < 0.05 ; **p < 0.01; ***p < 0.001; Scale bar = 200µm.

Supplementary Table 1

Antibody	Manufacturer	Catalogue number
Anti-B MyB (phospho T487)	Origene	TA301225
Anti-CD90.2	eBiosciences	11-0903
Anti-CD4	BD Pharmingen	17-0042
Anti-CD8	eBiosciences	553035
Anti-CD45R (B220)	BD Pharmingen	17-0452
Anti-Ly6C	eBiosciences	17-5932
Anti-Ly6G	eBiosciences	11-5931
Anti-CD115	eBiosciences	12-1152
Anti-CD11b	eBiosciences	47-0112
Anti-CD11c	eBiosciences	11-0114
Anti-mPDCA	Miltenyi	130-091-964
Anti-CD31	eBiosciences	11-0311-81
Anti-Ki-67	Thermo Scientific	RM-9106-S
Anti-Cleaved Caspase-3 (Asp175)	Cell signalling	#9579
Anti-CD274 (PD-L1, B7-H1)	eBioscience	12-5982
Anti-CD279 (PD-1)	eBioscience	11-9981
Anti-CD127 (IL-7Ra)	eBioscience	17-1271
Anti- CD25 (IL-2Ra)	eBioscience	12-0251
Anti-NK1.1	eBioscience	12-5941
Anti-CD3	eBioscience	11-0031

Supplementary Table 1: List of antibodies used for immunofluorescence. All antibodies listed were used in 1:100 dilutions to their original concentration for flow-cytometry and/or for immunohistochemistry.

Supplementary Table 2

Primer	number
Eukaryotic 18S RNA	#4333760T
GAPDH	Mm03302249_g1
Tipin	Mm00600456_m1
Atp6ap2	Mm00510396_m1
Atp6v0b	Mm01193846_g1
Pkmyt1	Mm01309244_m1
Arcn	Mm00524375_m1
Copb1	Mm00446330_m1
Copa	Mm00550231_m1
Mat2a	Mm00728688_s1
Eif3g	Mm00469383_m1
Eif3a	Mm00468721_m1
Arhgap23	Mm01722379_m1
MYBL2	Mm00485340_m1
Ifnar1	Mm00439544_m1
Ifnar2	Mm00494916_m1
TGFß	Mm01178820_m1
MMP14	Mm00485054_m1
MMP2	Mm00439498_m1
MMP9	Mm00442991_m1
PDGFß	Mm00440677_m1
VEGFA	Mm00437306_m1
VEGFB	Mm00442102_m1
VEGFC	Mm00437310_m1
VEGFD (Figf)	Mm01131929_m1
FGFR1	Mm00438930_m1
FGFR2	Mm01269930_m1
FGFR3	Mm00433294_m1
EGF	Mm00438696_m1
ANGPT1	Mm00456503_m1
SELL	Mm00441291_m1
CCL5	Mm01302427_m1
CXCL3	Mm01701838_m1
Csf3	Mm00438334_m1
CXCL15	Mm00441263_m1
CXCL1	Mm04207460_m1

Supplementary Table 2: List of murine Primers for Taqman qPCR
List of commercially available TaqMan® Gene Expression Assays from Life technologies used for detection of murine gene expression via qPCR.

Supplementary Table 3

Primer	number
Gapdh	QT01658692
IFNα4	QT01774353
IFNß	QT00249662
IRF7	QT00245266
OAS1	QT01056048
ISG15	QT02274335
Ly6C	QT00247604
CCR2	QT02276813
CCL2	QT00167832

Supplementary Table 3: List of murine Primers for SYBR Green-based qPCR
List of commercially available QuantiTect Primer Assays used for detection of murine gene expression via qPCR.

Supplementary Table 4

Primer	number
Eukaryotic 18S RNA	#4333760T
IFNAR1	Hs01066116_m1
IFNAR2	Hs01022059_m1
CCR2	Hs00704702_s1
ITGAM	Hs00167304_m1
SELP	Hs00927900_m1
CXCL9	Hs00171065_m1
MMP9	Hs00957562_m1
CD14	Hs02621496_s1
CD16	Hs04334165_m1
IRF7	Hs01014809_g1
IFNB1	Hs01077958_s1
OAS1	Hs00973635_m1
USP18	Hs00276441_m1
PRKRA	Hs00269379_m1

Supplementary Table 4: List of human Primers for Taqman qPCR
List of commercially available TaqMan® Gene Expression Assays from Life technologies used for detection of human gene expression via qPCR.

Supplementary Table 5

Primer	Sequence
LCMV WE NP Forward	5'- CAA AGT ATT CAC ACG GCA TGG A
LCMV WE NP Reverse	5'- TGG GAG AGC ACC TAT AAC TGA TGA

Supplementary Table 5: LCMV-NP Primer Sequences
